# Delayed Fluorescence by Triplet–Triplet Annihilation from Columnar Liquid Crystal Films

**DOI:** 10.1021/acsaelm.2c00432

**Published:** 2022-06-27

**Authors:** Larissa G. Franca, Paloma L. dos Santos, Piotr Pander, Marília G. B. Cabral, Rodrigo Cristiano, Thiago Cazati, Andrew P. Monkman, Harald Bock, Juliana Eccher

**Affiliations:** † Department of Physics, Durham University, South Road, Durham, DH1 3LE, United Kingdom; ‡ Departamento de Física, Universidade Federal de Santa Catarina, 88040900, Florianópolis, Santa Catarina, Brazil; § Faculty of Chemistry, Silesian University of Technology, Strzody 9, 44-100 Gliwice, Poland; ∥ Departamento de Química, Universidade Federal da Paraíba, CEP 58051-900, João Pessoa, Paraíba, Brazil; ⊥ Centre de Recherche Paul-Pascal, CNRS & Université de Bordeaux, 33600, Pessac, France; # Departamento de Física, Universidade Federal de Ouro Preto − UFOP, 35400-000, Ouro Preto, Minas Gerais, Brazil

**Keywords:** columnar liquid crystals, optical spectroscopy, triplet−triplet annihilation, delayed fluorescence, solution-processed OLED

## Abstract

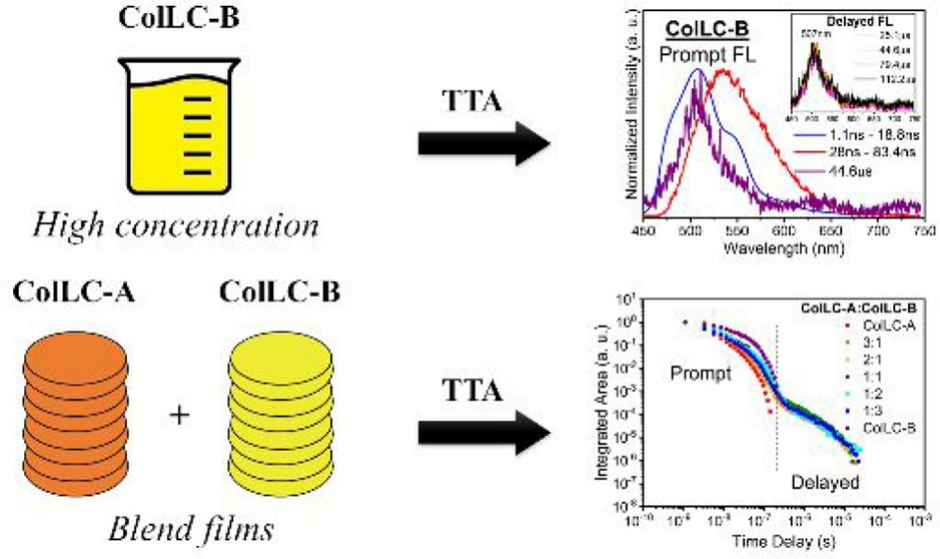

Delayed fluorescence (DF) by triplet–triplet annihilation (TTA) is observed in solutions of a benzoperylene-imidoester mesogen that shows a hexagonal columnar mesophase at room temperature in the neat state. A similar benzoperylene-imide with a slightly smaller HOMO–LUMO gap, that also is hexagonal columnar liquid crystalline at room temperature, does not show DF in solution, and mixtures of the two mesogens show no DF in solution either, because of collisional quenching of the excited triplet states on the imidoester by the imide. In contrast, DF by TTA from the imide but not from the imidoester is observed in condensed films of such mixtures, even though neat films of either single material are not displaying DF. In contrast to the DF from the monomeric imidoester in solution, DF of the imide occurs from dimeric aggregates in the blend films, assisted by the imidoester. Thus, the close contact of intimately stacked molecules of the two different species in the columnar mesophase leads to a unique mesophase-assisted aggregate DF. This constitutes the first observation of DF by TTA from the columnar liquid crystalline state. If the imide is dispersed in films of polybromostyrene, which provides an external heavy-atom effect facilitating triplet formation, DF is also observed. Organic light-emitting diodes (OLEDs) devices incorporating these liquid crystal molecules demonstrated high external quantum efficiency (EQE). On the basis of the literature and to the best of our knowledge, the EQE reported is the highest among nondoped solution-processed OLED devices using a columnar liquid crystal molecule as the emitting layer.

## 1 Introduction

Liquid crystals (LCs) have dominated display technology since the 1970s.^1^ However, in recent years, LC displays are being challenged by new technologies, which have received broad interest from the industry.^2^ As a consequence, the semiconductor properties of LCs started to play an important role, extending the application of LCs into optoelectronic devices, such as organic field effect transistors (OFETs),^3−5^ organic light emitting diodes (OLEDs),^6−11^ and organic solar cells (OSCs).^12−18^ In particular, columnar liquid crystals (ColLCs) comprise disk-like shaped molecules that are stacked into columns by strong π–π interactions.^19^ In these materials, anisotropic electronic transport occurs preferentially along the columns. Consequently, the molecular order in the intracolumnar packing is fundamental for high charge mobility.^20,21^ Furthermore, ColLCs have a propensity to self-healing of structural defects within the stack originating from their local fluidity. This property leads to a better charge transport, as such structural defects appear as a limiting factor of organic semiconductors.^22^ The ability to control molecular order and macroscopic alignment of ColLCs permits a significant improvement in device properties, because uniform anisotropic alignment of the molecular dipoles of the emissive layer may promote better light extraction in OLEDs and thus improved external quantum efficiency (EQE). Additionally, an improvement in charge transport properties can be obtained as the molecular alignment may induce a significant increase in charge mobility.^23−25^

Aromatic systems based on perylene derivatives are particularly interesting as they are easily functionalized, are thermally and chemically stable, and have high photoluminescence quantum yields.^26−30^ For this reason, the ColLC studied in this work consists of a planar π-conjugated core based on a perylene derivative and alkyl ester and imide groups forming the periphery of the conjugated disk. A more detailed discussion about the design of these molecules can be found in the original works.^31,32^

OLED efficiency is limited because of spin statistics, as triplet and singlet excited states form in a 3:1 ratio as a result of charge carrier recombination.^29^ In this situation, only singlet states are able to decay radiatively, and the other 75% of excited states remain unused (*dark*), leaving only 25% as upper limit for the internal quantum efficiency (IQE). The design of molecules exhibiting efficient phosphorescence,^30,33^ thermally activated delayed fluorescence (TADF),^34,35^ or delayed fluorescence (DF) from triplet–triplet annihilation (TTA) has emerged as a strategy to harvest dark triplet states.^36^ Phosphorescent OLED emitters often contain a heavy metal atom in their structure, which through inducing spin–orbit coupling strongly accelerates phosphorescence radiative rates.^37^ TADF molecules have a small energy gap between the lowest excited singlet and triplet states, enabling thermal up-conversion from the triplet state to the singlet manifold.^38,39^ TTA, alongside TADF, is an important triplet harvesting process in OLEDs. TTA molecules are able to achieve IQE of up to 65.2% in OLEDs.^38^ This mechanism involves the collision of two triplet excitons, which can fuse to give one singlet exciton. As a result of up-conversion of triplet excited states into singlets, delayed fluorescence (DF) can be observed. Efficient triplet diffusion and a high exciton concentration are very important for TTA to occur.^40^ Many materials exhibit TTA, among them polycyclic aromatic hydrocarbons, of which the best-known is pyrene.^41^ P-type delayed fluorescence is an older term for TTA and originates from its discovery in pyrene. To the best of our knowledge, DF by TTA has never been observed in liquid crystal forming molecules.

The aim of this work was to combine two perylene-based ColLCs to investigate their photophysical properties and evaluate their potential application in OLEDs. To our surprise, we observed a first example of a ColLC mesogen exhibiting delayed fluorescence in solution which manifestly occurs via the TTA mechanism. Even more significantly, we observed TTA DF also in thin liquid crystalline films of mixtures of the two mesogens, which is to our knowledge the first time that DF by TTA is observed in the columnar liquid crystalline state of matter, whereas TADF has recently been reported with columnar liquid crystalline carbazole-substituted terephthalonitriles bearing alkoxyphenyl substituents.^42^ DF is generally observed only either in solution, where collisions between fluorescent chromophores leading to radiationless triplet de-excitation are limited, or in the crystalline or glassy amorphous solid state, where intramolecular motions and collisions leading to radiationless de-excitation are frozen out. The observation of DF by TTA in the viscous fluid condensed mesophase indicates that the columnar state of the emitter mixture allows at the same time anisotropic chromophore alignment and bimolecular DF.

## 2 Experimental Section

### 2.1 General

Steady-state absorption and emission spectra in solution and thin film were acquired using an Ocean Optics spectrophotometer (Model USB4000) and a Hitachi fluorescence spectrophotometer (Model F-7000), respectively.

Time-resolved spectroscopy was performed using time-correlated single photon counting (TCSPC) and time-gated acquisition (iCCD) techniques. For TCSPC, a Picoquant modular fluorescence spectrometer (Model Fluotime 200) was used. The samples were excited using a 401 nm pulsed laser diode with repetition rates varying from 5.0 to 20 MHz. Lifetimes were obtained from a fluorescence decay by fitting with a multiexponential function using FluoFit 2.0 software. Time-resolved photoluminescence spectra were recorded using an ultrafast 4 PICOS iCCD camera (Stanford Computer Optics) with a pulsed (10 Hz) Nd:YAG laser (EKSPLA-SL312) excitation source at 355 nm.

### 2.2 Materials

**Figure 1:**
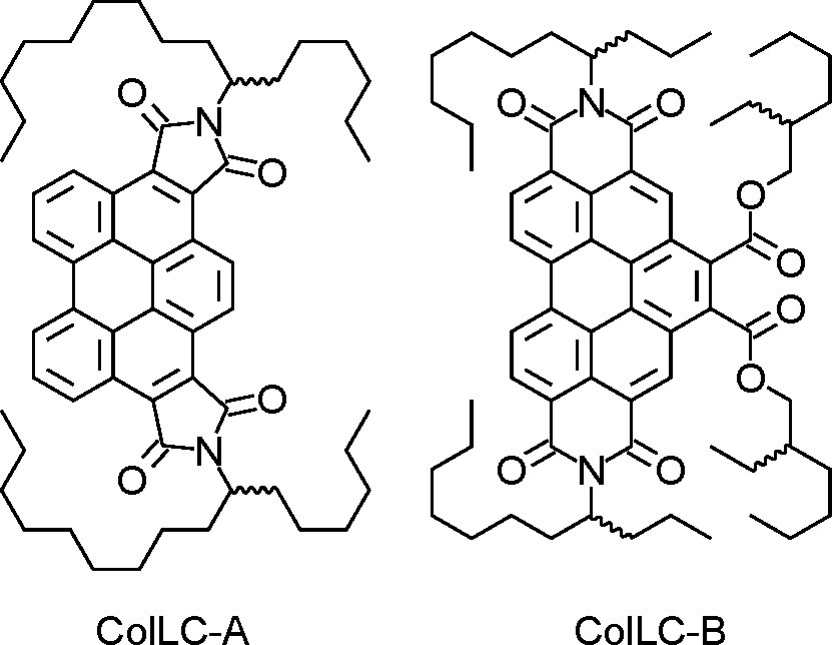
Chemical structures of **ColLC-A** (left, clearing temperature 265 °C) and **ColLC-B** (right, clearing temperature 151 °C), which both exhibit a hexagonal columnar mesophase at room temperature and up to the clearing transition.

The synthesis and characterization of the two room-temperature columnar liquid crystals (ColLCs) investigated in this work, *N*,*N*′-bis(1-hexyldecyl) benzo[*ghi*]perylene-3,4:11,12-tetracarboxdiimide (**ColLC-A**)^31^ and benzo[*ghi*]perylene-1,2,4,5,10,11-hexacarboxylic 1,2-bis(2-ethylhexyl)ester 4,5:10,11-bis(undec-4-yl)imide (**ColLC-B**),^32^ have been reported previously. Their chemical structures are shown in Figure 1. In addition, a detailed characterization of **ColLC-B** (coded as H4 in the original work) molecule has been published elsewhere.^23,24^

### 2.3 Calculations

DFT calculations were performed using Orca 4.2.1 software^43−45^ and visualized using Avogadro 1.2.0.^46,47^ Ground state geometry and molecular orbital (MO) iso surfaces were calculated using the B3LYP^48,49^ functional and the 6-31G**^50^ basis set. Excited state geometries were optimized with time-dependent density functional theory (TD-DFT) with Tamm-Dancoff approximation (TDA) using the same combination of functional and basis set. All optimized geometries were confirmed to be energetic minima through frequency calculation. All MO iso surfaces were rendered with an iso value of 0.03 if not stated otherwise. In order to simplify calculations, long alkyl chains were reduced to isopropyl or ethyl groups.

### 2.4 Sample Preparation

Stock solutions of individual compounds were prepared in chloroform with a concentration of 10 mg mL^–1^, and mixtures were produced in different ratios of **ColLC-A:ColLC-B** (3:1; 2:1; 1:1; 1:2; 1:3, v/v). For measurements in solution, the stock solutions were diluted to achieve concentrations of 0.17 mg mL^–1^ and 0.017 mg mL^–1^. Degassed solutions were obtained by 4 freeze–pump–thaw cycles to remove all dissolved oxygen.^51^ Neat and blend thin films of **ColLC-A/B** were produced from 10 mg mL^–1^ solutions and deposited by spin coating on glass or sapphire substrates at 2000 rpm for 30 s, followed by annealing at 50 °C for 30 min. Films in a polymer matrix were fabricated by spin-coating on sapphire substrates at 1000 rpm for 60 s. Films of **ColLC-A/B** in the two polymer matrices poly(4-bromostyrene) (4BrPS) and Zeonex^52^ were produced with 1% and 10% w/w concentrations of emitter from chloroform (4BrPs 100 mg mL^–1^) and toluene (Zeonex 100 mg mL^–1^) solutions, respectively.

## 3 Results and Discussion

### 3.1 Theoretical Calculations

**Figure 2:**
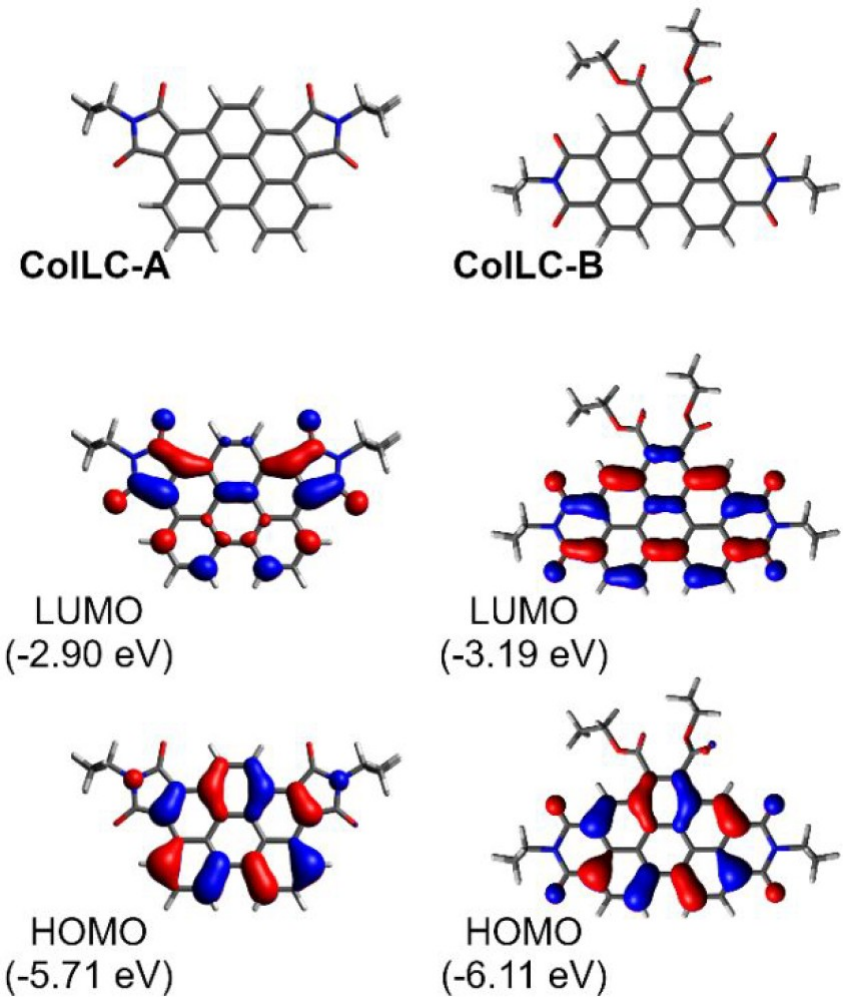
Optimized ground-state geometry (top) and HOMO/LUMO iso surfaces (middle and bottom). Alkyl groups are shortened to isopropyl and ethyl.

The calculated ground-state geometries (Figure 2) of **ColLC-A** and **ColLC-B** show a planar configuration of the aromatic central unit in both compounds. The planarity of the aromatic core of the molecules is favorable for the formation of the columnar liquid crystal mesophase, made of disordered stacks of disk-shaped molecules. HOMO and LUMOs are generally localized on the aromatic π-conjugated core with contributions from imide units in both compounds. The HOMO → LUMO transition shows a predominant π–π* character, which is typical for this class of molecules.^53,54^

To understand the T_1_ → S_1_ up-conversion pathway, the excited state energy landscape has been analyzed at the T_1_ geometry. The flat structure of both **ColLC-A** and **ColLC-B** does not leave room for significant changes in structural geometry of the molecule at different electronic states; thus, the T_1_ geometry can reasonably be considered similar to that of other excited states.

**Figure 3:**
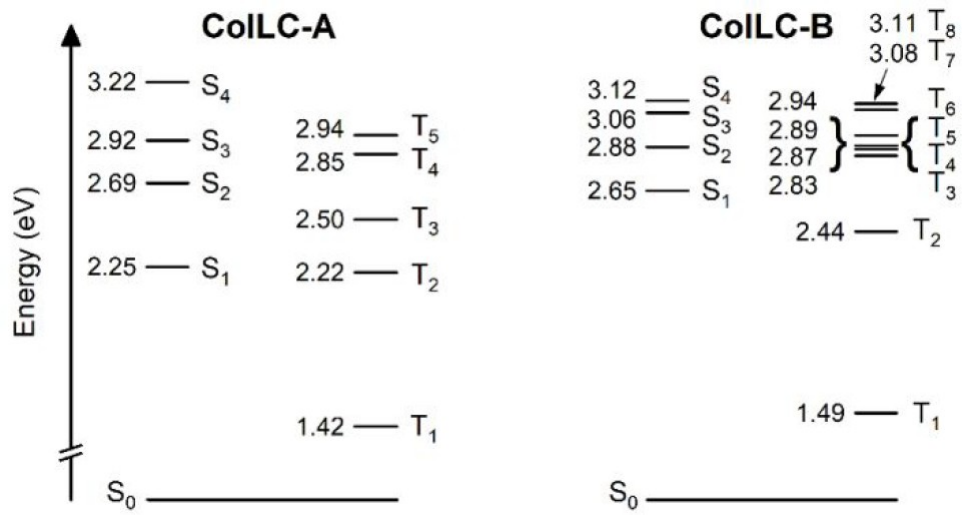
Excited-state energy diagram for **ColLC-A** and **ColLC-B** at the T_1_ geometry.

In **ColLC-A** and **ColLC-B**, both the excitations to S_1_ and T_1_ involve the HOMO → LUMO transition, thus giving them a π–π* character. In **ColLC-A** the S_1_ is at 2.25 eV and T_1_ is at 1.42 eV, giving a singlet–triplet energy gap Δ*E*_ST_ of 0.83 eV at the T_1_ geometry. In **ColLC-B** S_1_ is at 2.65 eV and T_1_ is at 1.49 eV, giving a Δ*E*_ST_ of 1.16 eV (Figure 3). The calculated T_1_ energy of both molecules suggests that their phosphorescence falls within the near-infrared region (i.e. ≈830–870 nm). These results are consistent with the expected behavior of such highly conjugated polycyclic diimides.^55^

In both systems, the direct transition from S_1_ to T_1_ is forbidden, as both states present the same orbital geometry. The large Δ*E*_ST_ of these compounds suppresses intersystem crossing; thus, low triplet formation yields are to be expected. These conditions are disallowing TADF occurring as a T_1_ → S_1_ up-conversion process because the Δ*E*_ST_ is large.^29,39^ Similarly, a large T_2_-T_1_ energy gap of 0.80–0.95 eV in both **ColLC-A** and **ColLC-B** disqualifies TADF mechanisms involving upper triplet states as intermediates. On the other hand, the S_1_ energy is less than double the T_1_ energy in both of the molecules, allowing for triplet up-conversion via TTA.

### 3.2 Photophysical Properties

#### 3.2.1 Optical Spectroscopy in Solution

**Figure 4:**
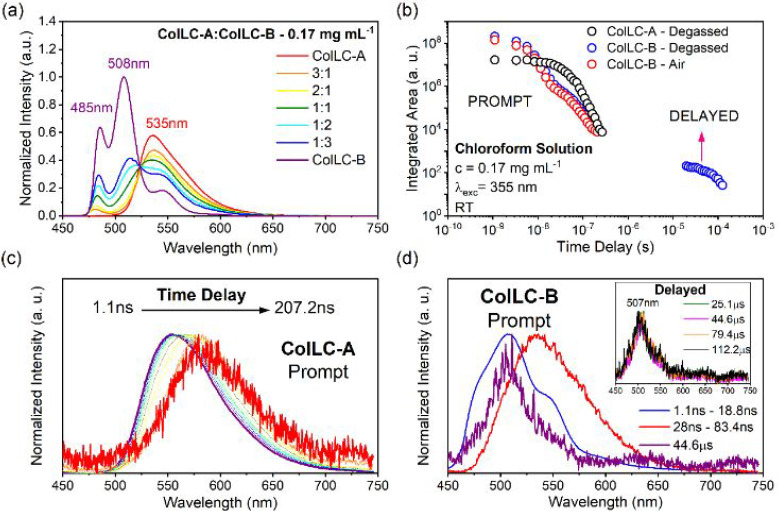
(a) Photoluminescence (PL) spectra of **ColLC-A** and **ColLC-B** recorded in individual solutions and mixtures (*c* = 0.17 mg mL^–1^). The spectra are normalized to the emission maximum of the most intense spectrum of **ColLC-B** at 508 nm, retaining their relative intensity (spectra non-normalized in Figure S10). (b) Time-resolved fluorescence decay curves of **ColLC-A** and **ColLC-B** in degassed and air-equilibrated solutions. Time-resolved emission spectra for (c) **ColLC-A** and (d) **ColLC-B** in degassed solutions at room temperature.

To evaluate the concentration dependence in the luminescent systems **ColLC-A** and **ColLC-B**, photophysical characteristics were investigated at two concentrations in chloroform: 0.017 mg mL^–1^ and 0.17 mg mL^–1^ which correspond to molar concentrations of ≈10^–5^ M and ≈10^–4^ M. We use the acronyms **ColLC-A** and **ColLC-B** to identify the two liquid crystal-forming molecules throughout the manuscript, including in solution, even though they do not present liquid crystal properties there. Steady-state absorption and emission spectra at concentration 0.017 mg mL^–1^ are shown in Figure S3. The mixtures show predominant characteristics of **ColLC-B** in both absorption and emission spectra as it has a higher extinction coefficient (Figure S3a). Excited-state lifetimes for **ColLC-A** and **ColLC-B** in chloroform solution at 0.017 mg mL^–1^ were obtained from TCSPC measurements. The photoluminescence of **ColLC-A** and **ColLC-B** decays monoexponentially with lifetimes of 11.0 and 2.3 ns, respectively (Figure S4). Moreover, both individual molecules, **ColLC-A** and **ColLC-B**, presented relatively high photoluminescence quantum yield (PLQY) of 64% and 60%, respectively, in chloroform solution. Figure 4a shows the photoluminescence (PL) characteristics of mixtures at 0.17 mg mL^–1^. Emission contributions in mixture solutions follow the trend in the ratio of emitters. A clear isoemissive point shows that the emission of the two materials are dependent on each other: The increasing quenching of the **ColLC-B** emission is accompanied by a proportional increase of the **ColLC-A** emission, as the amount of the latter is increased in the mixture. This quenching occurs because of Förster resonant energy transfer (FRET) between the two materials, as the absorption spectrum of **ColLC-A** overlaps with the fluorescence spectrum of **ColLC-B** (Figure S5).

Figure 4b shows decay traces of **ColLC-A** and **ColLC-B** in 0.17 mg mL^–1^ solutions. The decay curves of both molecules show biexponential behavior in the prompt fluorescence regime, indicating emission from two different species. The data also show a longer lived (delayed) emission for **ColLC-B** with a lifetime of around 58 μs (Figure S6). The longer lived emission is only present in degassed solution, but not in aerated solution. This DF is not observed in solutions of mixtures of **ColLC-A** and **ColLC-B** (Figure S7) which suggests that **ColLC-A** interferes with the triplet excited states of **ColLC-B** in solution (i.e., through collisional quenching).

DF of **ColLC-B** was not observed in a more dilute solution (0.017 mg mL^–1^, Figure S8), which indicates that the DF mechanism is bimolecular (i.e., by TTA).^56^ This is in agreement with calculations, as the modeled value of Δ*E*_ST_ = 1.16 eV in **ColLC-B** impedes TADF at room temperature. At low concentration, the **ColLC-B** molecules are further apart, thus reducing chances for collision of two triplet excited states with formation of the excited singlet state—TTA is thus unlikely in these conditions. However, TADF as an intramolecular process would not be suppressed by a low emitter concentration. In conclusion, we have demonstrated the TTA mechanism by varying the concentration of the emitter. TTA is highly dependent on emitter concentration as the important part of the mechanism involves intermolecular collisions—therefore, it should be present at high and absent at low concentrations. This approach is equivalent to recording power dependence at a fixed concentration.

Figure 4c shows the time-resolved PL spectra of **ColLC-A**, where a gradual redshift is observed in the nanosecond time regime. An isoemissive point in the time-resolved area-normalized emission spectra (Figure S9) indicates that the redshift observed is due to two emissive species (monomer and dimer) decaying independently of each other. Time-resolved spectra of a degassed solution of **ColLC-B** show a triexponential decay (Figure 4d). In the first regime, 1.1 to 18.8 ns, the emission peaks at 507 nm, which is attributed to molecular fluorescence. The second regime, 28 to 83.4 ns, shows featureless emission with a peak centered at 535 nm, which can be attributed to dimer fluorescence, as dimer emission is characterized by a broadened, featureless, and most importantly redshifted emission spectra.^57^ Emission spectra in the third (microsecond) time regime, 25.1 to 112.2 μs, are depicted in the inset of Figure 4d. These DF spectra match the emission in the fastest regime (<18.8 ns, purple line in Figure 4d) and can be attributed to the TTA mechanism. Despite the similarity in the molecular structure of both compounds, only **ColLC-B** showed DF in solution. This might indicate that the different types and locations of the peripheral substituents may influence the modes of the intermolecular interaction and consequently affect radiationless decay from T_1_.

#### 3.2.2 Optical Spectroscopy in the Condensed (Liquid Crystalline) State

**Figure 5:**
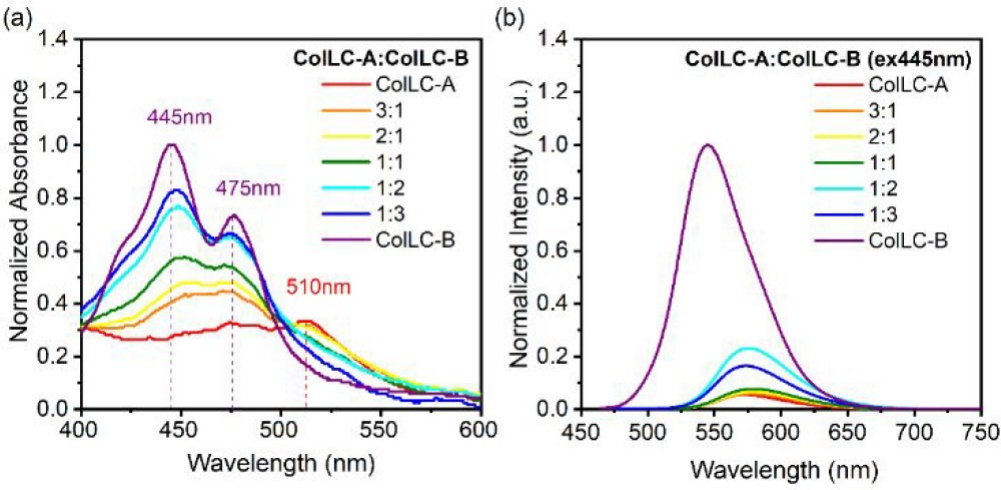
(a) Absorption and (b) emission spectra of neat and mixed films of **ColLC-A** and **ColLC-B**. The spectra are normalized to the emission maximum of the most intense spectrum of **ColLC-B** at 508 nm, retaining their relative intensity (spectra non-normalized in Figure S15).

A clear change in the absorption onset between the individual materials in solution (Figure S3a) and in condensed state spectra is observed, indicating dimer formation in the films. In the condensed state, the blended films presented simple sums of the absorption spectra of neat films of **ColLC-A** and **ColLC-B** (Figure 5a). All emission spectra are featureless, with a single maximum, suggesting that the emission is most likely occurring from dimer species (Figure 5b). Emission spectra of blends are almost exclusively from **ColLC-A**, at all ratios, despite the 445 nm excitation being absorbed by both components. A significant quenching in the emission intensity of **ColLC-B** with only a small amount of **ColLC-A** present in the blend suggests significant energy transfer from **ColLC-B** to **ColLC-A** in the condensed state, in agreement with measurements in solution. This indicates efficient FRET^57^ as there is an overlap between the absorption of **ColLC-A** and the photoluminescence of **ColLC-B** in the film (Figure S11).

In order to understand the high efficiency of FRET in **ColLC-A**:**ColLC-B** blends, we studied the liquid crystal properties of the mixtures. The differential scanning calorimetry (DSC) traces (Figure S12) show only one phase transition, which we interpret as clearing point from the columnar mesophase to the isotropic liquid state. The occurrence of only one phase transition in DSC of the mixtures indicates a full miscibility between **ColLC-A** and **ColLC-B** with the formation of a unique mesophase. In addition, the mesophase is stable down to room temperature in all cases. The type of the mesophase was confirmed by polarizing optical microscopy (POM) (Figure S13), which revealed typical textures of the hexagonal columnar mesophase formed by the two LCs at room temperature. The columnar order of the mesophase was verified by X-ray diffraction (Figure S14) from the appearance of the (10) and (20) peaks of the hexagonal columnar lattice. As discussed to DSC, the X-ray diffractograms also show no crystallization peak upon cooling down the clearing temperature to room temperature. The mesomorphic characterization of the blends suggests that the two ColLCs are fully miscible. This is in a good agreement with efficient FRET in the blends as the mechanism depends on the proximity of the two chromophores and therefore only remains active at relatively short distances. Furthermore, the liquid crystalline character of the blends persists at room temperature, being desirable for optoelectronic applications.

**Figure 6:**
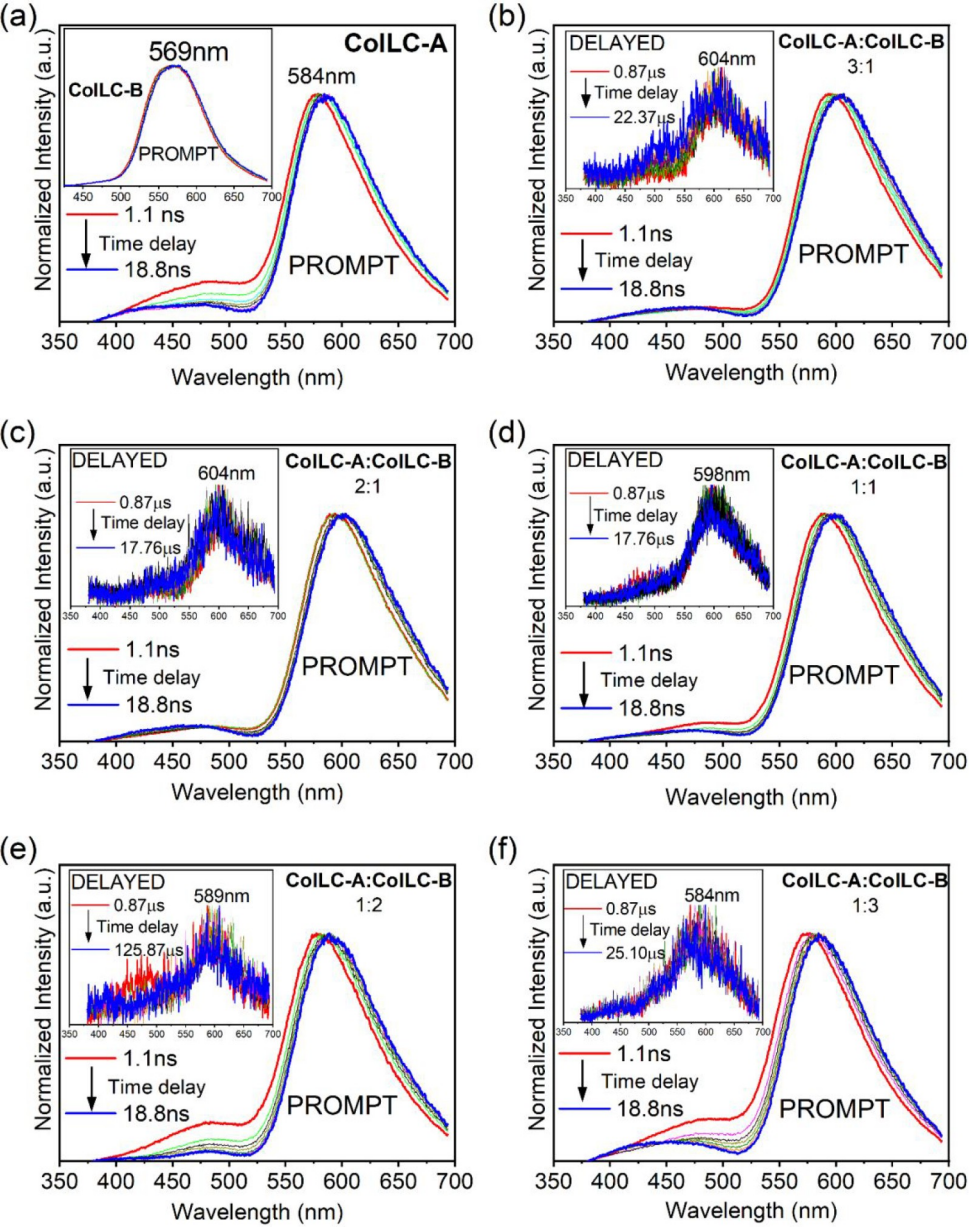
Time-resolved normalized emission spectra of: (a) **ColLC-A** and **ColLC-B** (inset). (b–f) Time-resolved normalized emission spectra of blend films, showing the prompt and delayed (insets) regimes. All measurements were performed at room temperature, using a 355 nm excitation source.

Time-resolved spectra for the single-component and blend films are shown in Figure 6. The neat film of **ColLC-A** presents a small redshift in emission over time delay (Figure 6a), indicating a distribution of dimer energy in the condensed state similar to that observed in solution (0.17 mg mL^−1^, Figure 4c). As seen in the inset of Figure 6a, the emission spectrum of **ColLC-B** is delay-independent in the whole investigated time range. Although **ColLC-B** shows DF in solution (0.17 mg mL^−1^), no DF is observed in neat films of either material. This confirms that in the condensed state, dimer formation quenches TTA in **ColLC-B** molecules. Probably because of the lower T_1_ energy, dimers appear to be quenching monomer triplet states, while dimer triplet states undergo accelerated nonradiative decay. On the other hand, in solution, TTA seems only to occur in monomer species by a collision-driven mechanism.

Figure 6b–f shows time-resolved emission spectra of blend films in the short delay region (attributed to prompt fluorescence), while the insets represent spectra recorded in the microsecond time range (delayed fluorescence). Within the first nanoseconds of delay, a small redshift is observed for all blends (Figure 6b–f), indicating a spread of **ColLC-A** dimer emission energy. At later times, the wavelength of maximum emission does not change, even in the microsecond time scale. There is also a small contribution from monomer emission of **ColLC-A** in the nanosecond regime in the wavelength range of 400–525 nm. However, in the 1:2 and 1:3 blend films (Figure 6e,f), this emission contribution can be attributed to the **ColLC-B** monomer (Figure S3c).

**Figure 7:**
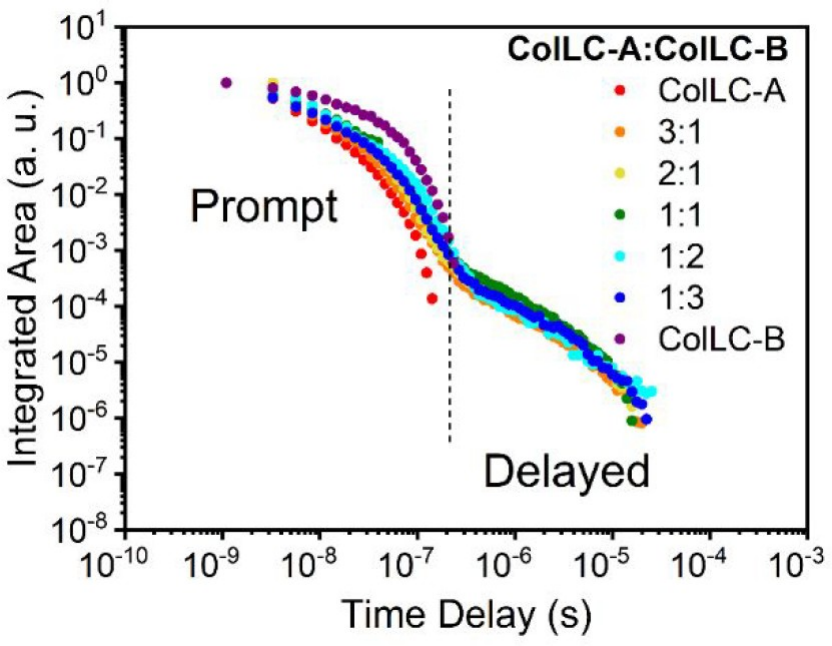
Time-resolved photoluminescence decay curves for **ColLC-A**, **ColLC-B**, and blend films at room temperature.

The decay curves for both the neat and blend films are presented in Figure 7. Blend films show two regimes: prompt and delayed fluorescence, while the single component films only show prompt fluorescence and lack any long-lived emission. A multiexponential expression (Figure S16 and Table S3) was used to fit the photoluminescence decay in the films. The average lifetime of DF of the blend films is in the range of ≈2–3 μs. The lifetimes are longer when the contribution of **ColLC-B** in the blend increases, and a maximum of 3.33 μs is obtained for the 1:2 ratio. This suggests that by increasing the contribution of **ColLC-B** molecules in the blend, the available triplet population increases, which favors TTA. However, a higher proportion of **ColLC-B** molecules leads to suppression of dimer formation between two **ColLC-A** molecules. As neither **ColLC-A** nor **ColLC-B** undergo TTA on their own in pristine films, and as **ColLC-A** does not show TTA on its own in solution (Figure 4), we conclude that its dimer form does TTA only upon provision of a sufficient quantity of triplet excited states. We note the **ColLC-B** molecule appears to have sufficient triplet formation yield or smaller T_1_ nonradiative decay rate than **ColLC-A** and shows TTA on its own in solution. Triplet excitons created on **ColLC-B** have a long lifetime, and so can migrate to a **ColLC-A** dimer where they become trapped. When trapped triplet excitons on **ColLC-A** dimer sites are close enough to each other, TTA can occur (Scheme S1). Consequently, the combination of both compounds in the blend films provides the necessary requirement for TTA to occur: elevated triplet formation yield in **ColLC-A** due to Dexter transfer from **ColLC-B** and dimer formation of **ColLC-A**. **ColLC-B** however does not show TTA in films, likely due to faster decay of its dimer triplets compared to its monomer triplets. It is worth noting here that only the **ColLC-B** monomer shows TTA in solution, but the dimer form does not, confirming that the exciton migration mechanism is different in the condensed state. The two pure materials and their mixtures are shearable between two glass plates at room temperature (Figure S17). These shearing experiments demonstrate that the condensed state retains fluidity of the mesophase at room temperature. This indicates that the residual mobility of the molecules in the film enables the TTA mechanism that relies on molecular encounters, while the viscosity hinders collision-induced radiationless de-excitation.

In order to further probe the origins of delayed fluorescence in blend films, an additional investigation was made using polymer hosts blended with either of the columnar materials. Time-resolved measurements were performed in the films of **ColLC-A** and **ColLC-B** doped in polymer matrices at two different concentrations: 1% wt. and 10% wt. Zeonex and poly(4-bromostyrene) (4BrPS) were chosen as two polymer matrices. Zeonex as an aliphatic (light atoms only) polymer can be considered neutral, while 4BrPS is a polymer bearing bromine atoms, thus inducing an external heavy atom effect. The 4BrPS was previously used to increase the triplet formation yield.^58^ An external heavy atom effect originating from the bromine atom is responsible for the increase in the intersystem crossing rates of the dopant molecules, thus it leads to an increased population of triplet states originating from optical excitation. The results of this study are presented in the Supporting Information (Figures S18 and S19). Only **ColLC-A** in 4BrPS at 10% concentration shows a delayed fluorescence component, while no DF is observed in any of the other seven films. This behavior is in line with the properties in **ColLC-A**:**ColLC-B** blend films described above. In contrast to its liquid solution, **ColLC-B** does not show TTA on its own in 1% loaded polymer films, presumably because the molecules are restricted and show low mobility, thus reducing chances of intermolecular interactions. On the other side, at 10% load **ColLC-A** exhibits dimer emission, while **ColLC-B** dimers were already shown not to present TTA. Consequently, **ColLC-A** only shows TTA in conditions which provide both: small distances between molecules facilitating dimer formation and provision of triplet states. These conditions are present in the 10%-loaded 4BrPS film.

To evaluate the potential application of the TTA phenomenon in ColLCs, OLEDs comprising blend films were fabricated (Figure S20 and S21). Electroluminescence spectra are shown in Figure S21d. Results and additional discussion of OLED performance are given in section 3 of the Supporting Information. Nondoped OLED devices using **ColLC-A** as the emitter present a high EQE of 1.8% with its electroluminescence maximum at 646 nm. This unexpectedly high EQE might be an indication of the TTA mechanism being at work in the **ColLC-A** OLED device as the initial triplet populations originating from electrical excitation are considerably larger than in optical measurements. On the basis of the literature and to the best of our knowledge (see Table S5 for details), we believe this EQE figure to be the highest among nondoped solution-processed OLED devices using columnar liquid crystal as the emitting layer. The OLEDs are still subject to optimization in the future; however, they provide a proof of concept for the feasibility of such systems in luminescent devices.

## 4 Conclusion

At a low concentration in solution, both individual compounds show photoluminescence from their monomer species. At an elevated concentration, dimer/excimer emission occurs because of strong intermolecular π–π interactions. In concentrated solution, **ColLC-B** shows DF, while **ColLC-A** only shows prompt fluorescence. The strong concentration dependence of the DF from **ColLC-B** indicates that it originates by a TTA mechanism. In the viscous condensed state, surprisingly, DF is only observed in blend films. This is due to the complementary functions of the two materials in these mixtures: TTA in the film occurs only from **ColLC-A** dimers; however these species do not generate enough triplets (or not sufficiently long-lived triplets) for TTA to appear on its own. **ColLC-B** provides triplet states for TTA to occur, but its dimers/excimers do not display TTA on their own. The combination of the two molecules in blends yields TTA from **ColLC-A** dimers fueled by the triplet population on **ColLC-B** dimers. Likewise, the external heavy atom effect of a bromopolymer matrix fuels emission from **ColLC-A**. The observation of delayed fluorescence in the condensed viscous fluid state of liquid crystal materials, where molecules can be uniformly oriented by annealing, opens up the possibility to use such materials as emissive layers of organic light-emitting diodes to enhance light outcoupling as well as charge and exciton transport.

## Supporting Information Available

Additional experimental details, materials, and methods, including photographs of the experimental setup, DFT calculations, additional photophysical and devices measurements, supplementary figures and discussions (PDF)

## ABBREVIATIONS

DF: delayed fluorescence
TTA: triplet–triplet annihilation
HOMO: highest occupied molecular orbital
LUMO: lowest occupied molecular orbital
OLEDs: organic light-emitting diodes
EQE: external quantum efficiency
LCs: liquid crystals
OFETs: organic field effect transistors
OSCs: organic solar cells
ColLCs: columnar liquid crystals
IQE: internal quantum efficiency
TADF: thermally activated delayed fluorescence
TCSPC: time correlated single photon counting
DFT: density functional theory
MO: molecular orbital
TD-DFT: time-dependent density functional theory
TDA: Tamm-Dancoff approximation
Δ*E*_ST_: singlet–triplet energy gap
PL: photoluminescence
FRET: Förster resonant energy transfer
4BrPS: poly(4-bromostyrene)
DSC: differential scanning calorimetry
POM: polarizing optical microscopy
