# Supplementary material for: Delayed Fluorescence by Triplet–Triplet Annihilation from Columnar Liquid Crystal Films
Source: ACS Appl Electron Mater. 2022 Jun 27;4(7):3486–94. doi: 10.1021/acsaelm.2c00432 (PMC9330766; doi:10.1021/acsaelm.2c00432)
Supplement: Supplementary file 1 — el2c00432_si_001.pdf [file el2c00432_si_001.pdf]

Supporting information:

Delayed fluorescence by triplet-triplet annihilation from columnar liquid crystal films

Larissa G. Franca[a,b *], Paloma L. dos Santos[a], Piotr Pander[c,a], Marília G. B. Cabral[d,e], Rodrigo Cristiano[d], Thiago Cazati[f], Andrew P. Monkman[a], Harald Bock[e] and Juliana Eccher [b *]

AUTHOR ADDRESS:

[a] Department of Physics, Durham University, South Road, Durham, DH1 3LE, United Kingdom

[b] Departamento de Física, Universidade Federal de Santa Catarina, 88040900, Florianópolis, SC, Brazil

[c] Centre for Organic and Nanohybrid Electronics, Silesian University of Technology, Konarskiego 22B, 44-100 Gliwice, Poland

[d] Departamento de Química, Universidade Federal da Paraíba, CEP 58051-900, João Pessoa, Paraíba, Brazil

[e] Centre de Recherche Paul-Pascal, CNRS & Université de Bordeaux, 33600, Pessac, France

[f] Departamento de Física, Universidade Federal de Ouro Preto – UFOP, 35400-000, Ouro Preto, MG, Brazil

Corresponding Author

*Larissa G. Franca (larissa.gomes-franca@durham.ac.uk).
*Prof. Juliana Eccher (juliana.eccher@ufsc.br).

Table of Contents

1 Calculations **2**

2 Additional measurements **5**

2.1 Optical spectroscopy in solution: *5*

2.2 Optical spectroscopy in the condensed (liquid crystalline) state: *10*

2.3 ***ColLC-A/B*** *thin films in polymers matrix:* *16*

2.4 Proposed mechanism for TTA in the blend films of ***ColLC-A:ColLC-B****:* *17*

3 OLEDs **18**

3.1 Methods *18*

3.2 Results and discussion: *19*

4 References **23**

1 Calculations

Table S1: Excited state energy at the T_1 geometry – molecule **ColLC-A**.

State	Energy, eV	Transition	Dominating character
S_1	2.25	HOMO→LUMO (90%)	π-π*
S_2	2.69	HOMO→LUMO+1 (76%)<br>HOMO-1→LUMO (21%)	π-π*
S_3	2.92	HOMO-1→LUMO (65%)<br>HOMO→LUMO+1 (17%)<br>HOMO→LUMO+3 (13%)	π-π*
S_4	3.22	HOMO-4→LUMO (83%)<br>HOMO-5→LUMO+1 (15%)	n-π*
T_1	1.42	HOMO→LUMO (95%)	π-π*
T_2	2.22	HOMO→LUMO+1 (73%)<br>HOMO-1→LUMO (16%)	π-π*
T_3	2.50	HOMO-1→LUMO (82%)<br>HOMO→LUMO+1 (15%)	π-π*
T_4	2.85	HOMO-2→LUMO (50%)<br>HOMO→LUMO+2 (33%)	π-π*
T_5	2.94	HOMO-4→LUMO (72%)<br>HOMO-5→LUMO+1 (22%)	n-π*

Table S2: Excited state energy at the T_1 geometry – molecule **ColLC-B**.

State	Energy, eV	Transition	Dominating character
S_1	2.65	HOMO→LUMO (91%)	π-π*
S_2	2.88	HOMO-1→LUMO (73%)<br>HOMO→LUMO+1(23%)	π-π*
S_3	3.06	HOMO-2→LUMO (95%)	n-π*
S_4	3.12	HOMO-3→LUMO (95%)	n-π*
T_1	1.49	HOMO→LUMO (96%)	π-π*
T_2	2.44	HOMO-1→LUMO (91%)	π-π*
T_3	2.83	HOMO→LUMO+1 (24%)<br>HOMO-9→LUMO (19%)<br>HOMO-10→LUMO (18%)	π-π*
T_4	2.87	HOMO-7→LUMO (27%)<br>HOMO-5→LUMO (14%)<br>HOMO→LUMO+3 (18%)<br>HOMO→LUMO+2 (11%)	π-π*
T_5	2.89	HOMO-9→LUMO (87%)	π-π*
T_6	2.94	HOMO-3→LUMO (89%)	n-π*
T_7	3.08	HOMO-4→LUMO (90%)	π-π*
T_8	3.11	HOMO→LUMO+1 (60%)<br>HOMO→LUMO+2(12%)	π-π*

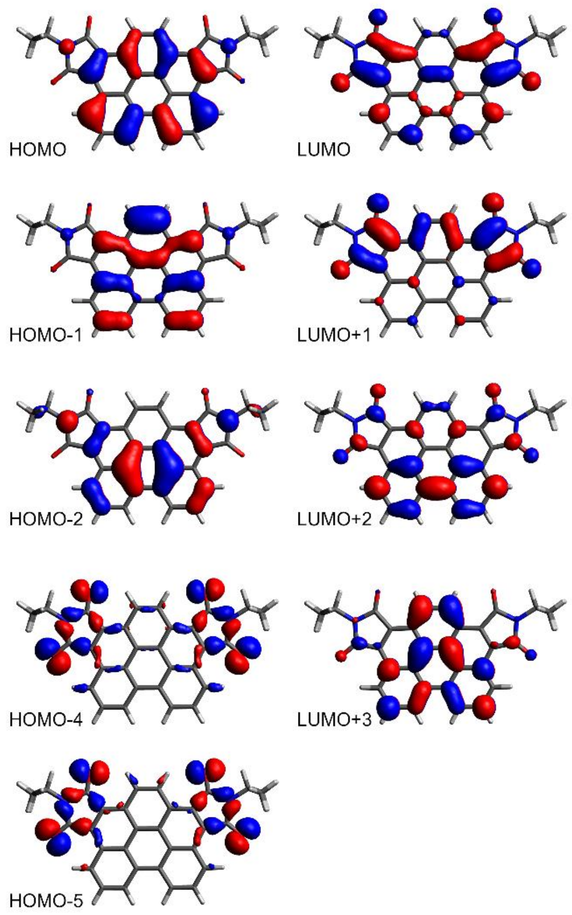

Figure S1: Relevant MO iso surfaces of **ColLC-A** at the T_1 geometry. Alkyl groups were shortened to isopropyl.

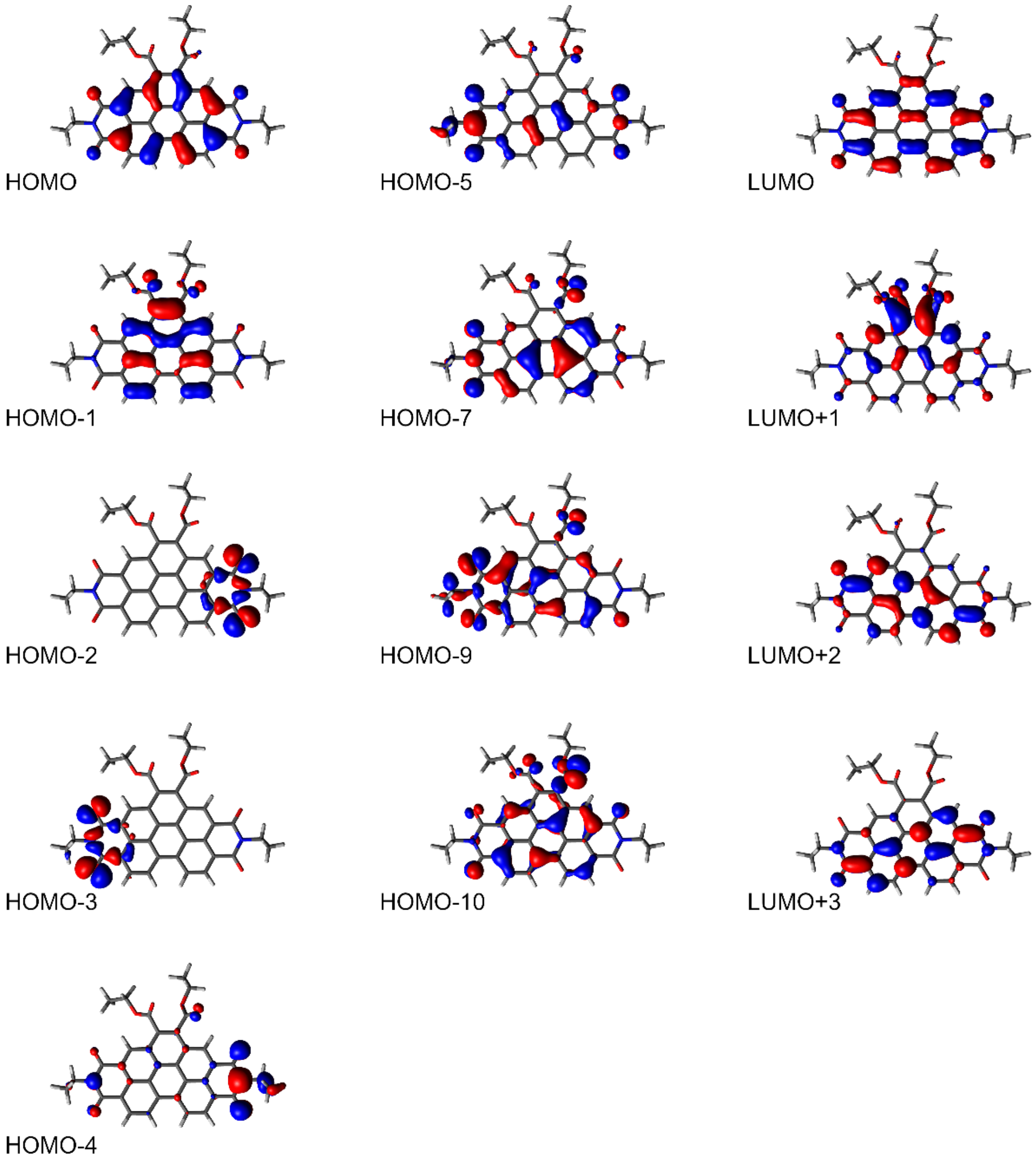

Figure S2: Relevant MO iso surfaces of **ColLC-B** at the T_1 geometry. Alkyl groups were shortened to isopropyl or ethyl.

2 Additional measurements

2.1 *Optical spectroscopy in solution:*

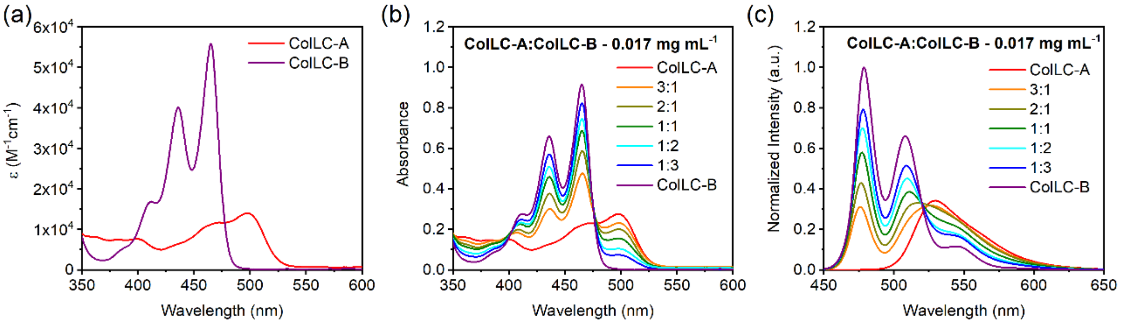

Figure S3: (a) Molar extinction coefficient of **ColLC-A/B** in chloroform solutions. (b) Absorption and (c) photoluminescence spectra of **ColLC-A** and **ColLC-B** for individual molecules and mixtures in chloroform solutions at 0.017 mg mL^{-1}, using a 401 nm excitation.

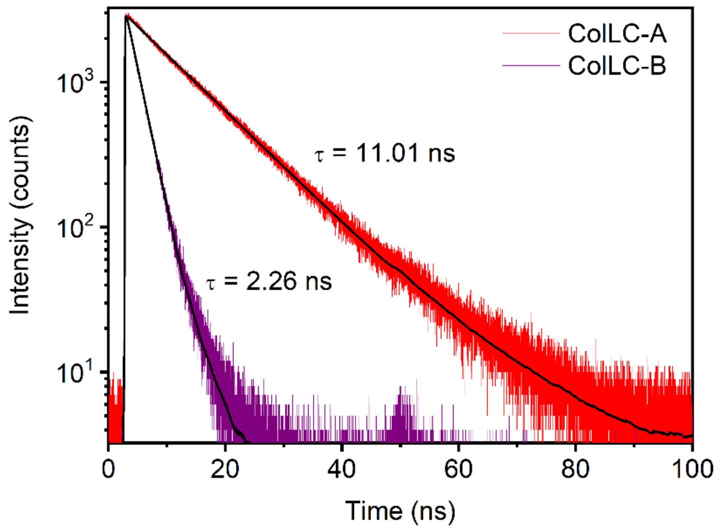

Figure S4: Photoluminescence decays of **ColLC-A** and **ColLC-B** in chloroform solutions at a concentration of 0.017 mg mL^{-1}. Decays were collected at 530 nm for **ColLC-A** and at 478 nm for **ColLC-B**, using 401 nm excitation and fitted using monoexponential fuction.

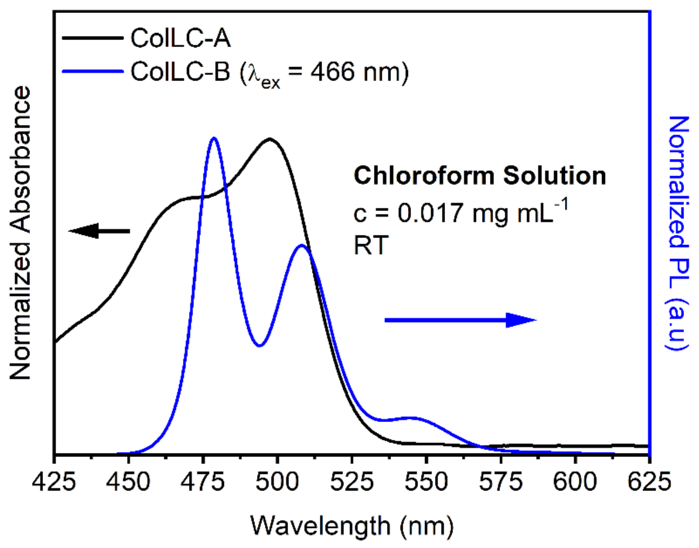

Figure S5: Spectral overlap between the emission of **ColLC-B** and absorption spectrum of **ColLC-A** in solution at a concentration of 0.017 mg mL^{-1}.

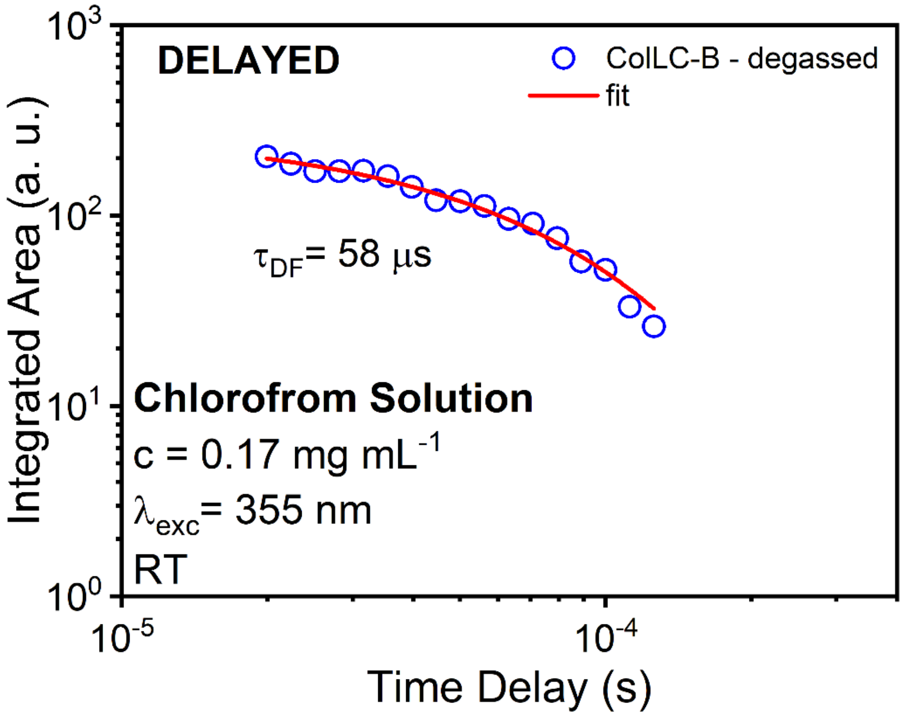

Figure S6: Time-Resolved fluorescence decay (microsecond region) of **ColLC-B** in solution at a concentration of 0.17 mg mL^{-1}. The data is fitted using a monoexponential fuction.

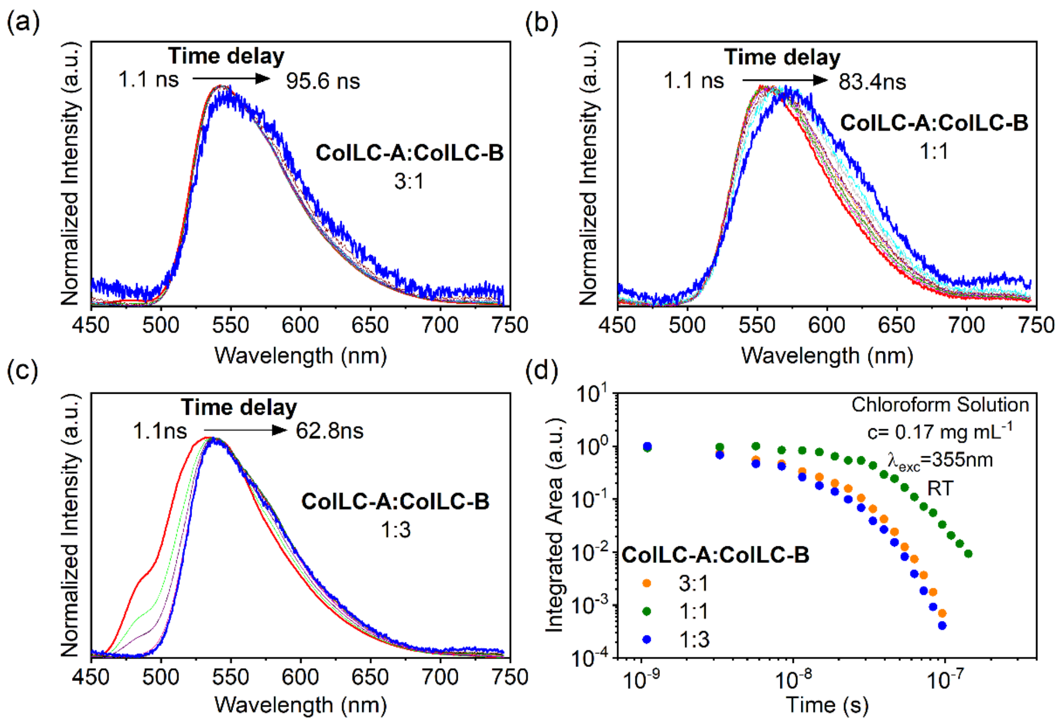

Figure S7: Time resolved normalized emission spectra of mixtures of **ColLC-A:ColLC-B** in the ratio of (a) 3:1; (b) 1:1 and (c) 1:3 v/v in chloroform solutions at 0.17 mg mL^{-1}. All measurements were performed in degassed solutions at room temperature, using a 355 nm excitation source. (d) Time resolved fluorescence decay curves in the entire temporal region of analyses.

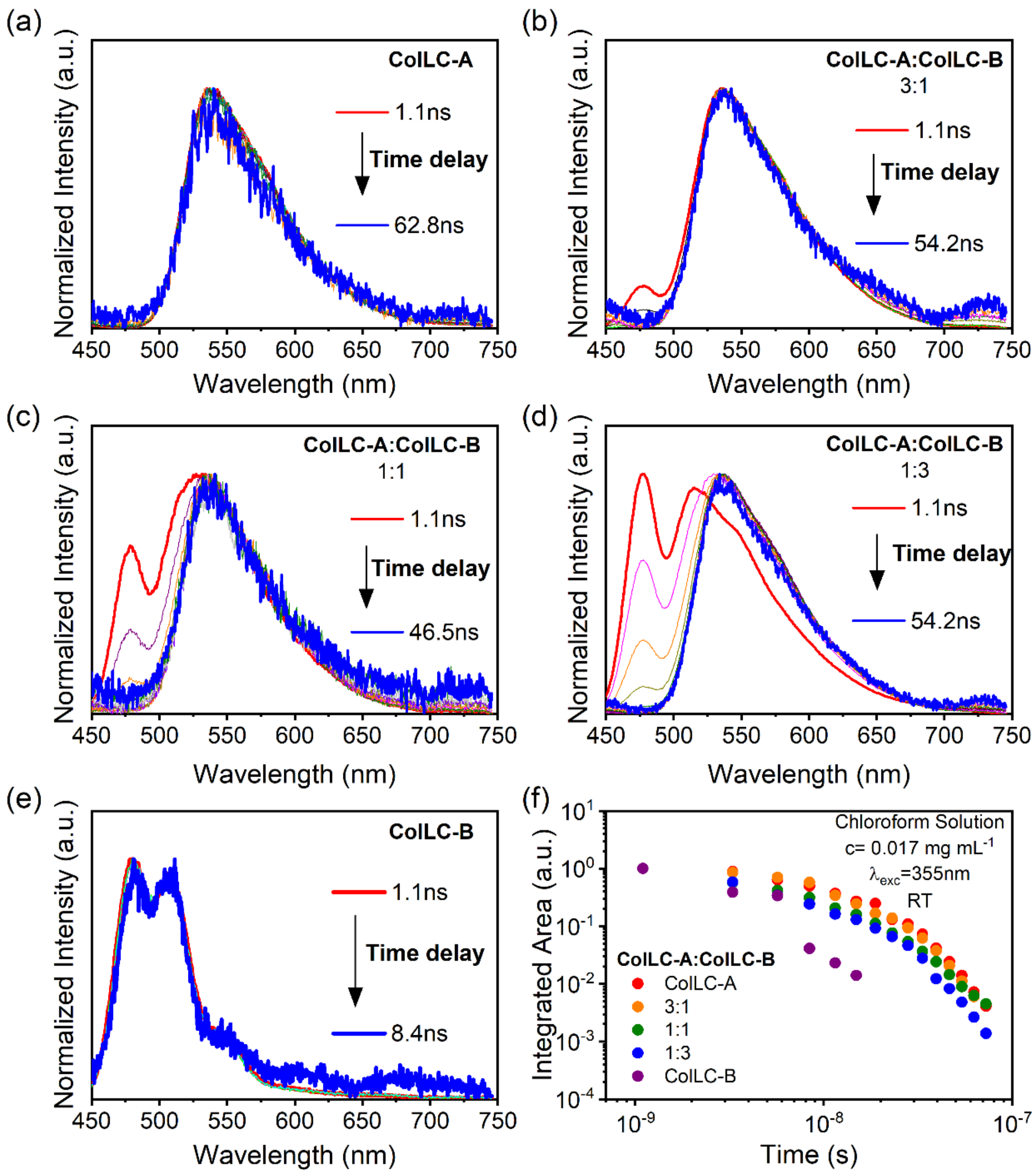

Figure S8: Time resolved normalized emission spectra of: (a) **ColLC-A**, mixtures of **ColLC-A:ColLC-B** in the ratio of (b) 3:1; (c) 1:1; (d) 1:3 v/v and (e) **ColLC-B** in chloroform solutions at 0.017 mg mL^{-1}. All measurements were performed in degassed solutions at room temperature, using a 355 nm excitation source. (f) Time resolved fluorescence decay curves in the entire temporal region of analyses.

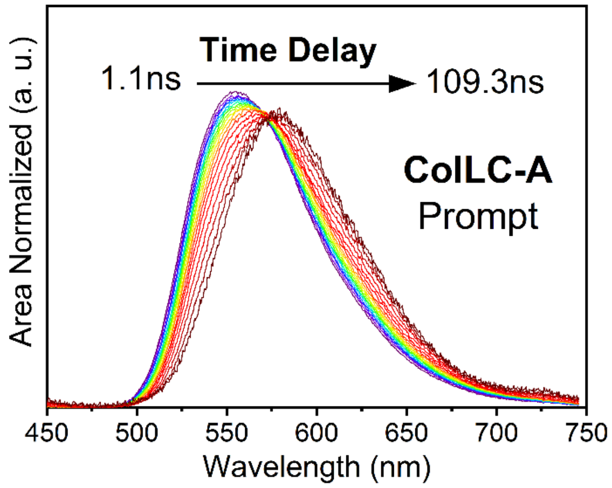

Figure S9: Area normalized time resolved emission decay of **ColLC-A** in chloroform solution at a concentration of 0.17 mg mL^{-1}, using a 355 nm excitation source.

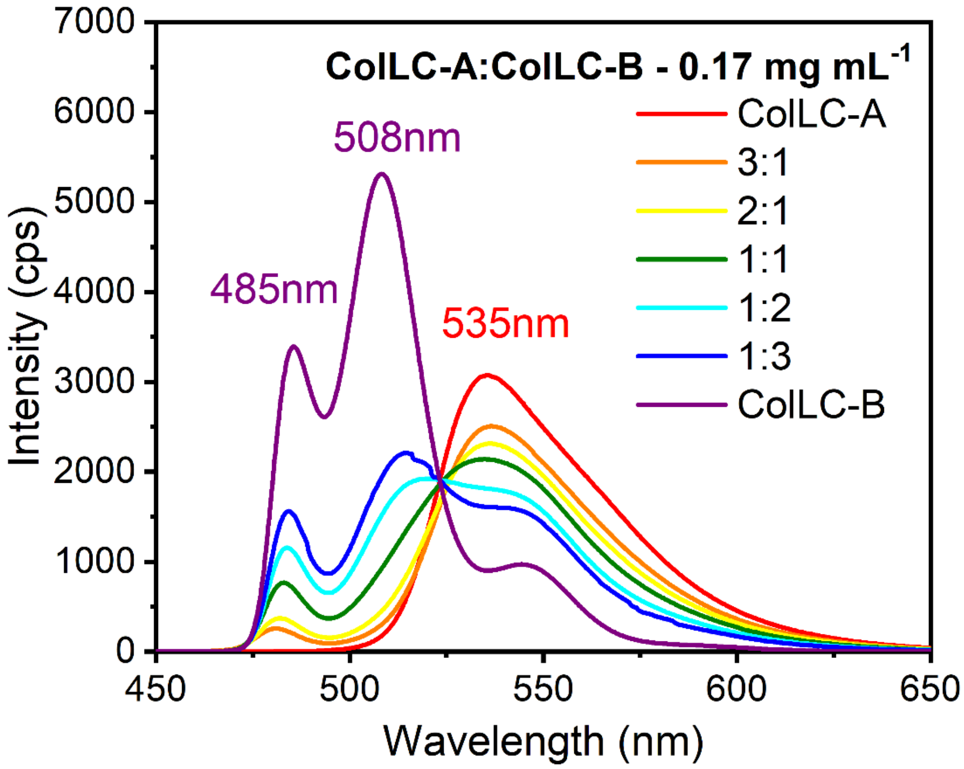

Figure S10: Non-normalized photoluminescence (PL) spectra of **ColLC-A** and **ColLC-B** of isolated molecules and mixtures in chloroform solutions at 0.17 mg mL^{-1}.

2.2 *Optical spectroscopy in the condensed (liquid crystalline) state:*

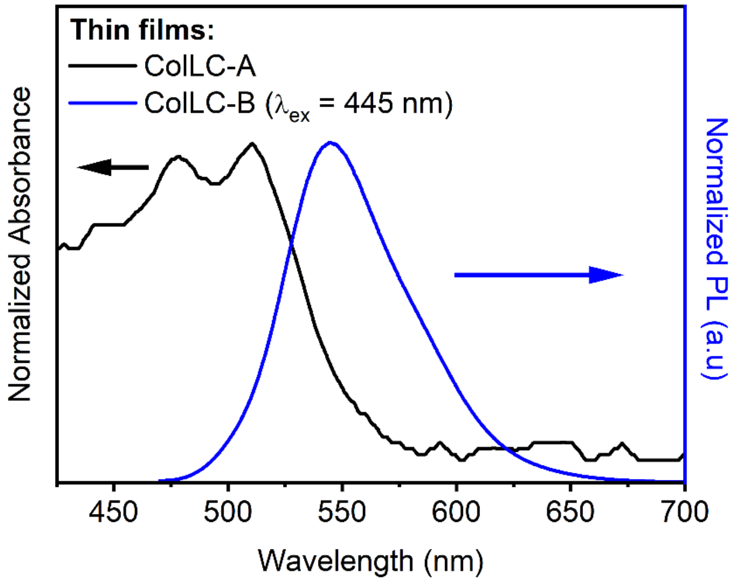

Figure S11: Spectral overlap between the emission of **ColLC-B** and absorption spectrum of **ColLC-A** in film.

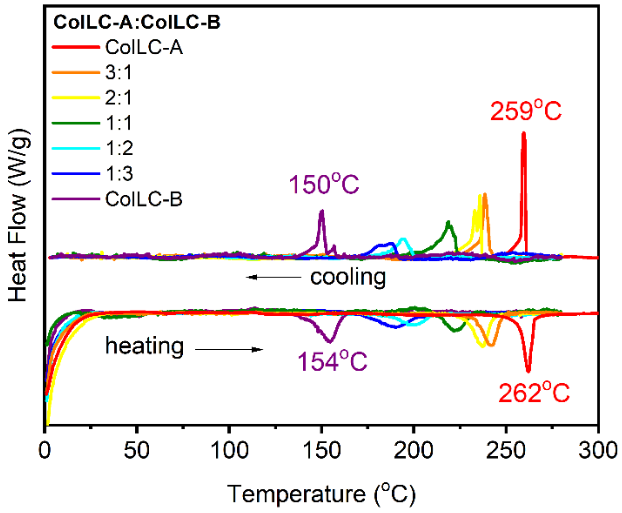

Figure S12: DSC traces for **ColLC-A**, mixtures of **ColLC-A:ColLC-B** in the ratio of 3:1; 2:1; 1:1; 1:2; 1:3 wt/wt and **ColLC-B**. The measurements were performed using a rate of 10 °C min^{-1}.

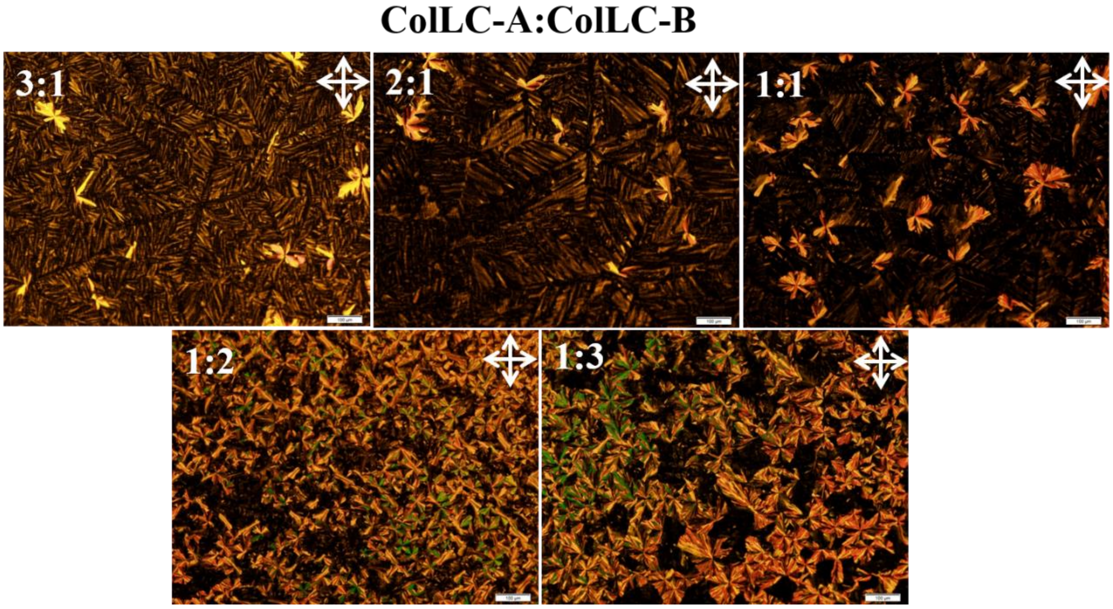

Figure S13: Optical images (100× magnification) obtained from polarizing optical microscopy of **ColLC-A:ColLC-B** in the ratio of 3:1; 2:1; 1:1; 1:2 and 3:1 wt/wt. Optical images were obtained at room temperature after cooling down from isotropic liquid state with a rate of 10 °C min^{-1}. The textures were taken of the sample confined between two glass slides under crossed polarizers.

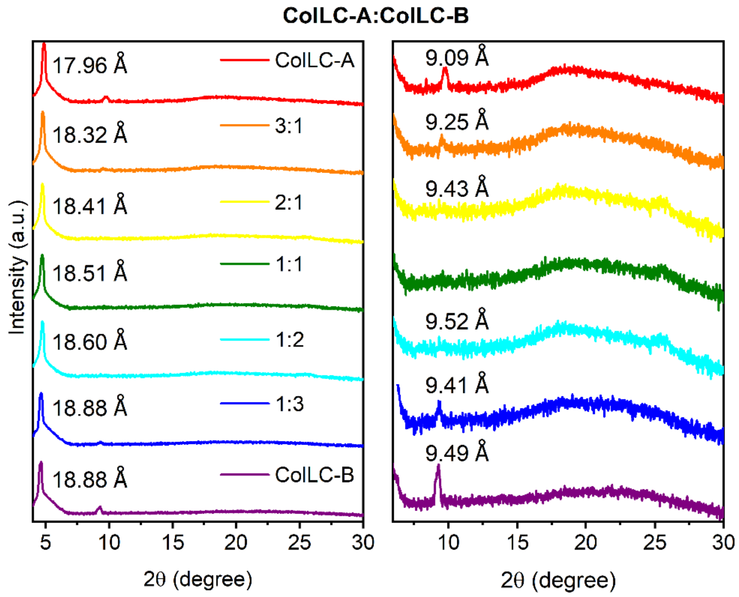

Figure S14: X-ray diffractograms at room temperature of drop casting films of **ColLC-A**, mixtures of **ColLC-A:ColLC-B** in the ratio of 3:1; 2:1; 1:1; 1:2; 1:3 v/v and **ColLC-B**.

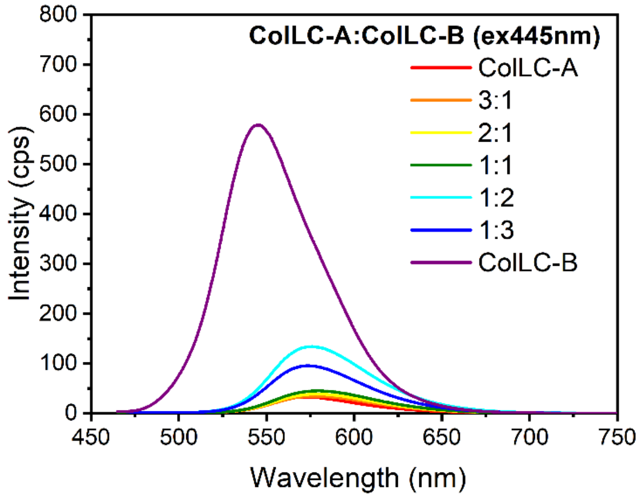

Figure S15: Non-normalized photoluminescence (PL) spectra of neat and mixed films of **ColLC-A** and **ColLC-B**.

Table S3: Excited state lifetimes and photoluminescence quantum yield (Φ_{PL}) of **ColLC-A**, **ColLC-B** and mixture films at room temperature. The lifetime data were obtained from the decay curves obtained with iCCD measurements by fitting with a multiexponential expression (**Figure S16**). The measurements of Φ_{PL} were performed in air-saturated environment, using a 505 nm excitation for **ColLC-A** and 445 nm excitation for **ColLC-B** and mixture films.

Films ColLC-A:ColLC-B	**ColLC-A**	**3:1**	**2:1**	**1:1**	**1:2**	**1:3**	**ColLC-B**
**Prompt FL**							
**t_1 (ns)**	2.1	4.5	4.5	4.2	6.1	2.9	5.5
**A_1 (%)**	70	67	73	74	86	79	47
**t_2 (ns)**	7.5	19.1	20.7	25.2	39.1	23.3	36.7
**A_2 (%)**	22	32	26	25	13	20	53
**t_3 (ns)**	22.9	73.8	70.0	65.2	89.6	75.5	-
**A_3 (%)**	8	1	1	1	1	1	-
**t_{av} (ns)**	11.4	18.3	17.0	21.0	25.5	19.8	33.0
**Delayed FL**							
**t_1 (µs)**	-	0.53	0.65	0.61	0.83	0.60	-
**A_1 (%)**	-	79	79	86	79	76	-
**t_2 (µs)**	-	3.66	4.07	4.20	4.92	3.84	-
**A_2 (%)**	-	21	21	14	21	24	-
**t_{av} (µs)**	-	2.57	2.79	2.54	3.33	2.77	-
**Φ_{PL} (%)**	17	10	16	16	18	17	37

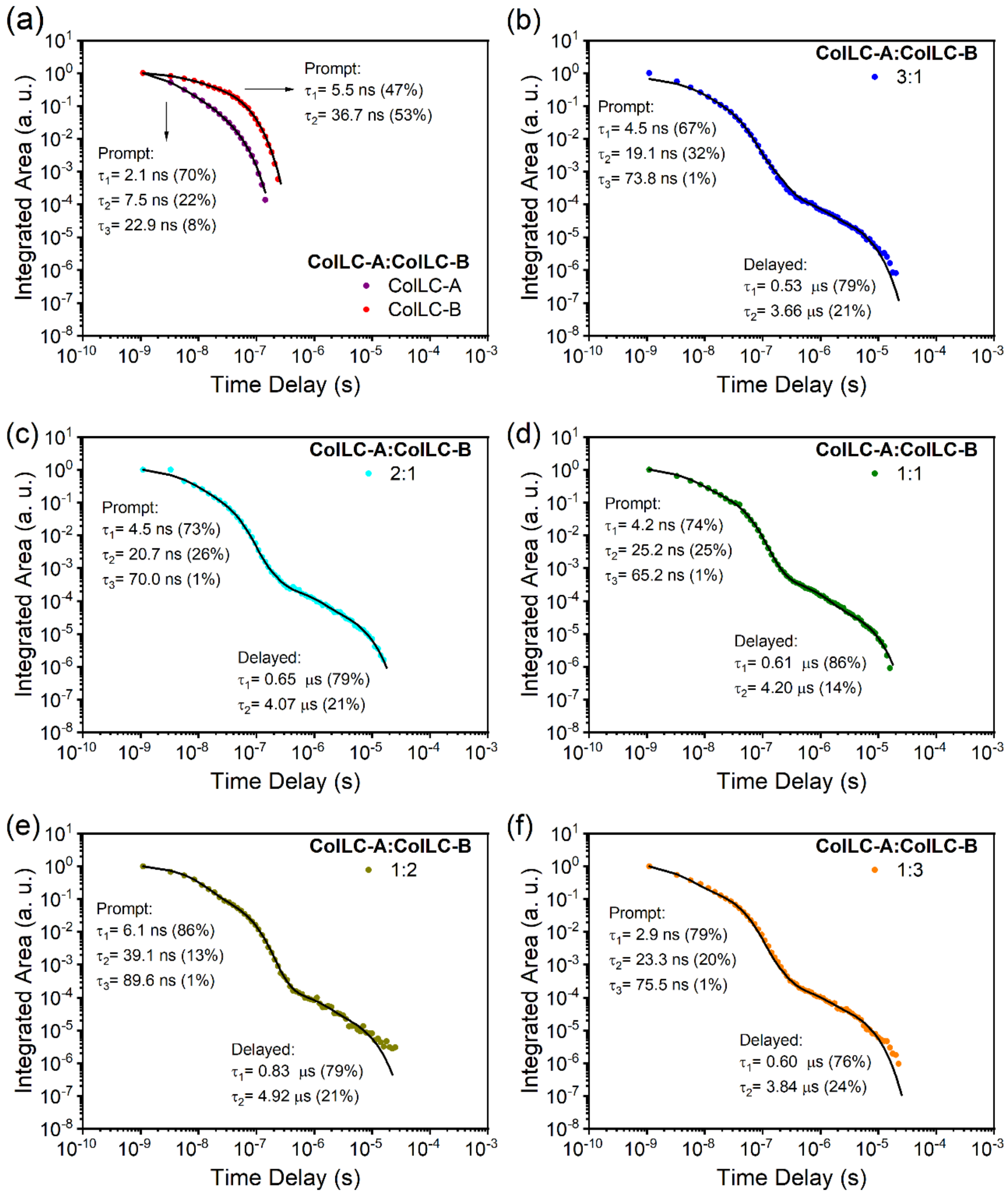

Figure S16: Time-Resolved fluorescence decays of: (a) **ColLC-A** and **ColLC-B** neat films, mixtures of **ColLC-A:ColLC-B** in the ratio of (b) 3:1; (c) 2:1; (d) 1:1; (e) 1:2; (f) 1:3 m/m at room temperature. The data are fitted using a multi-exponential function.

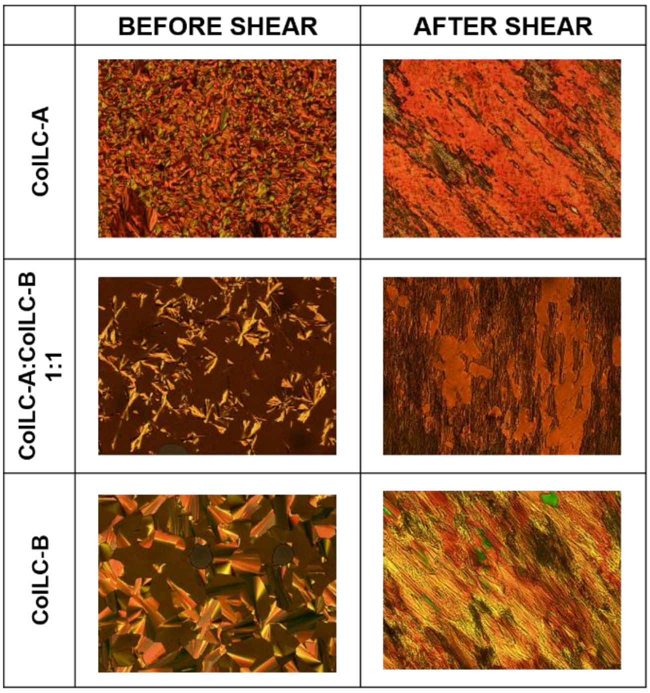

Figure S17: Optical images (200× magnification) obtained from polarizing optical microscopy of **ColLC-A**, **ColLC-A:ColLC-B** in the ratio of 1:1 wt/wt and **ColLC-B** at room temperature. In left panel: optical images before shearing between two glasses plates. In right panel: optical images after shearing at room temperature. Images obtained with slightly uncrossed polarizers.

2.3 ***ColLC-A/B** thin films in polymers matrix:*

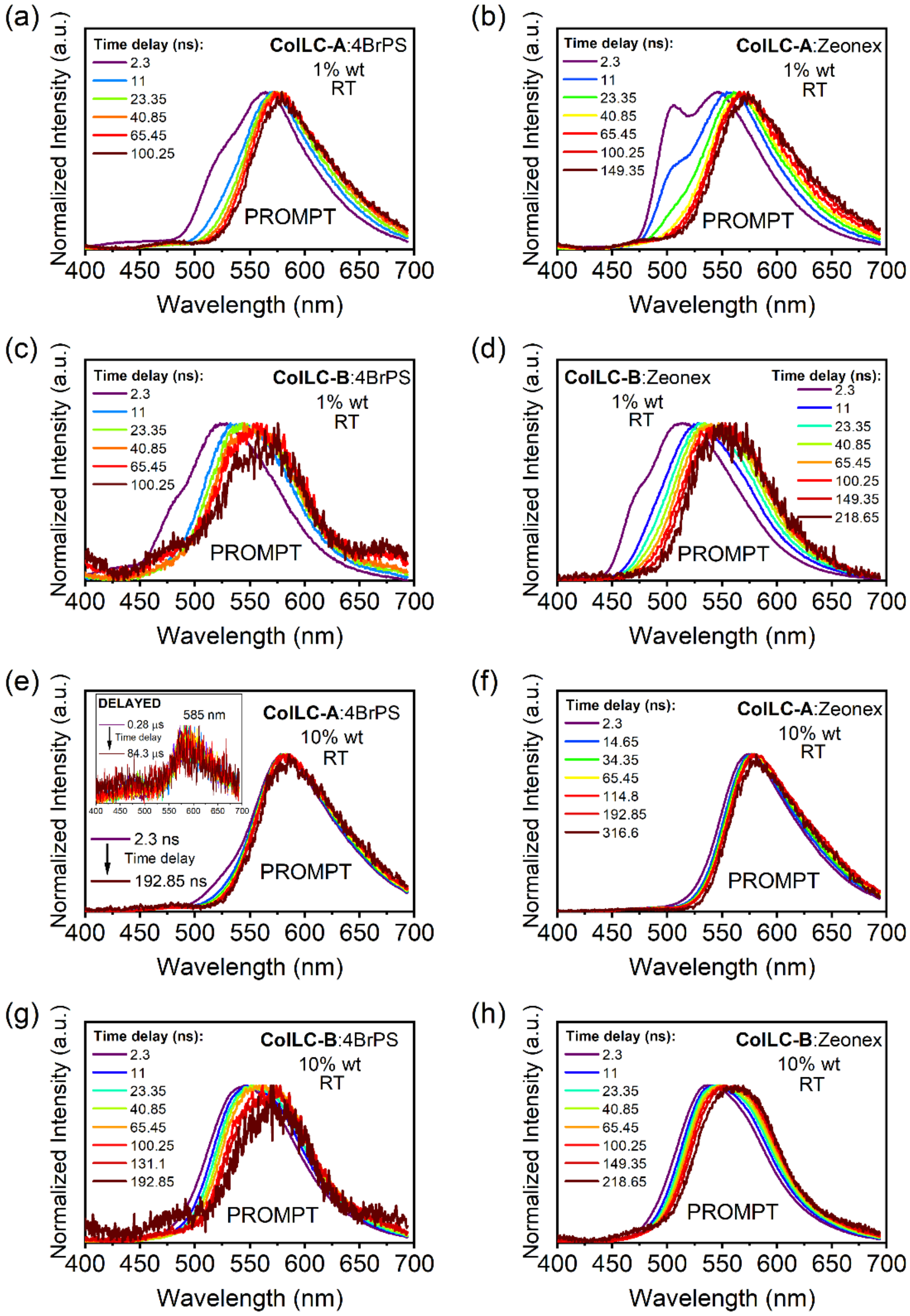

Figure S18: Time-resolved normalized emission spectra of **ColLC-A/B** in polymer matrices at two different concentrations (1%wt and 10%wt). (a) **ColLC-A**:4BrPS (1%wt), (b) **ColLC-A**:Zeonex(1%wt), (c) **ColLC-B**:4BrPS (1%wt), (d) **ColLC-B**:Zeonex (1%wt), (e) **ColLC-A**:4BrPS (10%wt, inset: delayed component), (f) **ColLC-A**:Zeonex (10%wt), (g) **ColLC-B**:4BrPS (10%wt) and (h) **ColLC-B**:Zeonex (10%wt).

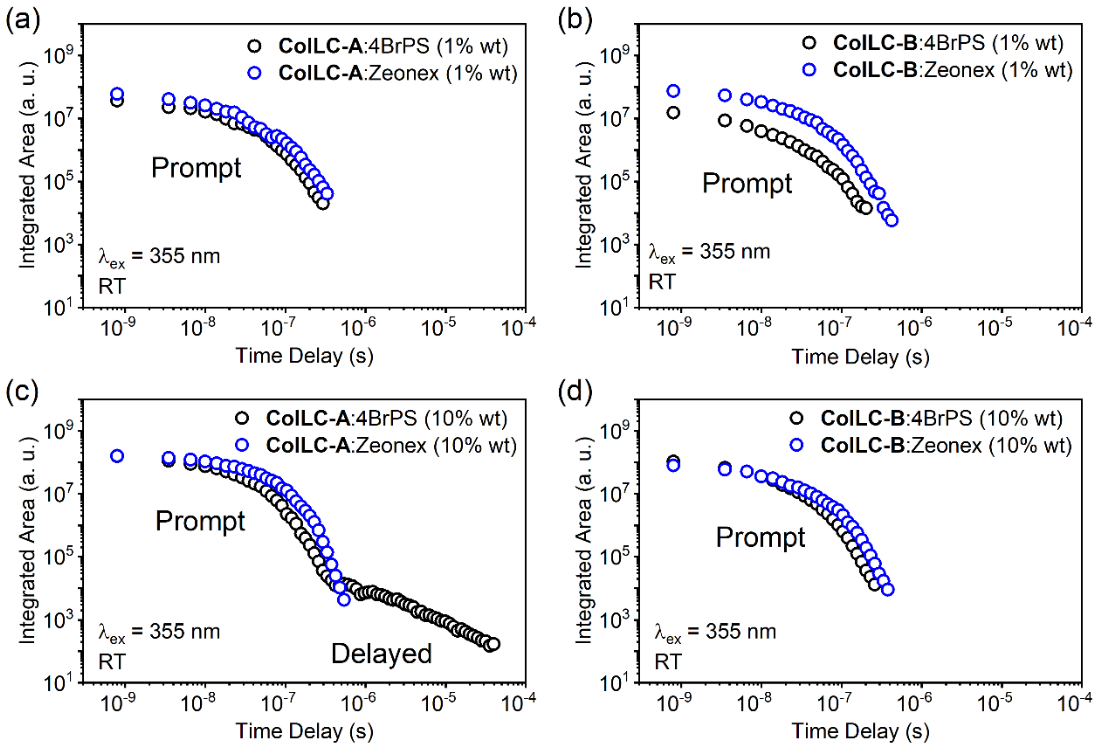

Figure S19*:* Time resolved fluorescence decay curves at room temperature in polymer matrices. (a) **ColLC-A**:polymer at 1%wt concentration, (b) **ColLC-B**:polymer at 1%wt concentration, (c) **ColLC-A**:polymer at 10%wt concentration and (d) **ColLC-B**:polymer at 10%wt concentration.

*2.4 Proposed mechanism for TTA in the blend films of **ColLC-A:ColLC-B**:*

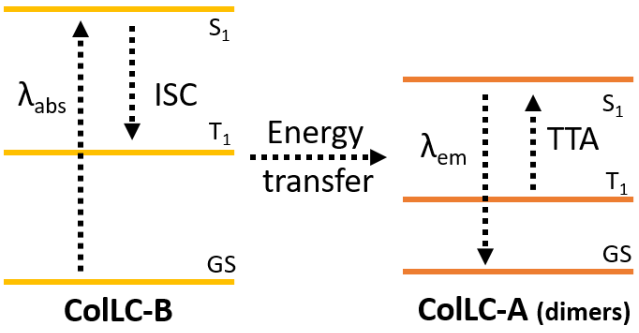

Scheme S1: Proposed mechanism for the TTA in the blend films of **ColLC-A:ColLC-B.**

3 OLEDs

3.1 Methods

OLEDs were fabricated by spin-coating / evaporation hybrid method. The hole injection layer (PEDOT AL4083), hole transport layer PVKH, and emitting layer (EML) were spin-coated, whereas the electron transport layer (PO-T2T) and cathode (LiF/Al) were evaporated. Devices of 4x4 mm, 4x2 mm, and 2x2 mm pixel size were fabricated. 2,4,6-Tris[3-(diphenylphosphinyl)phenyl]-1,3,5-triazine (PO-T2T, LUMTEC), poly(*N*-vinylcarbazole) (PVKH, Sigma Aldrich, M = 10^6 Da), LiF (99.995%, Sigma Aldrich), and Aluminium pellets (99.9995%, Lesker) were purchased from the companies indicated in parentheses. OLED devices were fabricated using pre-cleaned with ozone plasma indium-tin-oxide (ITO) coated glass substrates with a sheet resistance of 20 Ω cm^{-2} and ITO thickness of 100 nm. PEDOT AL4083 was spun-coated and annealed on a hotplate at 120 ˚C for 15 min to give a 30 nm film. PVKH layer was spun from chloroform:chlorobenzene (95:5 v/v) (3 mg mL^{-1}) while emitting layer was spun from toluene (10 mg mL^{-1}). PVKH layer was annealed at 50 ˚C for 5 min before depositing another layer. All solutions were filtered directly before application using a PVDF (organic solvents) and PES (PEDOT AL4083) syringe filter with 0.45 µm pore size. All other electron transport and cathode layers were thermally evaporated using Kurt J. Lesker Spectros II deposition system at 10^{-6} mbar base pressure. All organic materials and aluminium were deposited at a rate of 1 Å s^{-1}. The LiF layer was deposited at a rate of 0.1–0.2 Å s^{-1}. Characterisation of OLED devices was conducted in a 10 inch integrating sphere (Labsphere) connected to a Source Measure Unit Keithley 2400 and coupled with a spectrometer USB4000 (Ocean Optics). Further details are available in reference[1].

3.2 Results and discussion:

ColLC-A and **ColLC-B** as well as their mixtures were incorporated into solution-processed OLEDs to demonstrate their potential applicability as emissive layers. The device structure used was: ITO | Al4083 (30 nm) | PVKH (12 nm) | EML (30 nm) | PO-T2T (50 nm) | LiF (0.8 nm) | Al (100 nm). Achieving low barrier for charge injection into the emissive layer was rather challenging due to the low HOMO (-6.1 in **ColLC-A** and -6.6 in **ColLC-B**) and LUMO (-3.4 in **ColLC-A** and -3.7 in **ColLC-B**) energy of the emitters[2,3]. Thus, despite the use of an additional hole transport layer (PVKH) as well as electron transport layer (PO-T2T) with low LUMO, the V_{ON} in all cases was at least 10 V (**Table S3**). Given the HOMO being deeper in **ColLC-B** than in **ColLC-A** we observe a gradual increase of V_{ON} with the rising contribution of the former in the EML. As a result the device based on 100 % **ColLC-B** did not yield any luminescence and its characteristics could not be recorded. We have tried another device structure, using molybdenum(VI) oxide instead of PEDOT:PSS: ITO | MoO_x (10 nm) | PVKH (12 nm) | **ColLC-B** (30 nm) | PO-T2T (50 nm) | LiF (0.8 nm) | Al (100 nm) – in this case it was possible to record the electroluminescence spectrum of the device (**Figure S15d**), however the electroluminescent characteristics of it could not be recorded.

The electroluminescence spectrum of devices involving **ColLC-A** as the emitter, especially 100% **ColLC-A** demonstrate electroluminescence spectra matching the photoluminescence of the compound in film. On the other side, electroluminescence of the device with EML composed of 100 % **ColLC-B** is most likely an interfacial exciplex at the boundary with PVKH layer. **ColLC-B** has strong electron-accepting properties and is likely to form exciplex with PVKH (HOMO ~ -5.4 eV), while the electroluminescence spectrum is significantly more red shifted than photoluminescence in neat film. OLEDs show relatively high external quantum efficiency (EQE) among other liquid crystal forming materials (**Table S4**), especially device involving 100% **ColLC-A** in the EML with 1.8 %. A relatively low brightness of OLEDs is due to their high V_{ON} and the voltage limitation of the power supply, 20 V. The maximum brightness of 100% **ColLC-A** device is likely to reach 1000-2000 cd m^{-2} at > 20 V. We will continue to work on the device structure to further decrease V_{ON} and improve device efficiency, but the presented structures provide a proof-of-concept that the liquid crystal forming emitters presented in this work can achieve high OLED efficiency, comparable with the best known for this category of molecules. We believe that upon further optimisation of the molecular structure a further improvement towards highly efficient devices can be achieved.

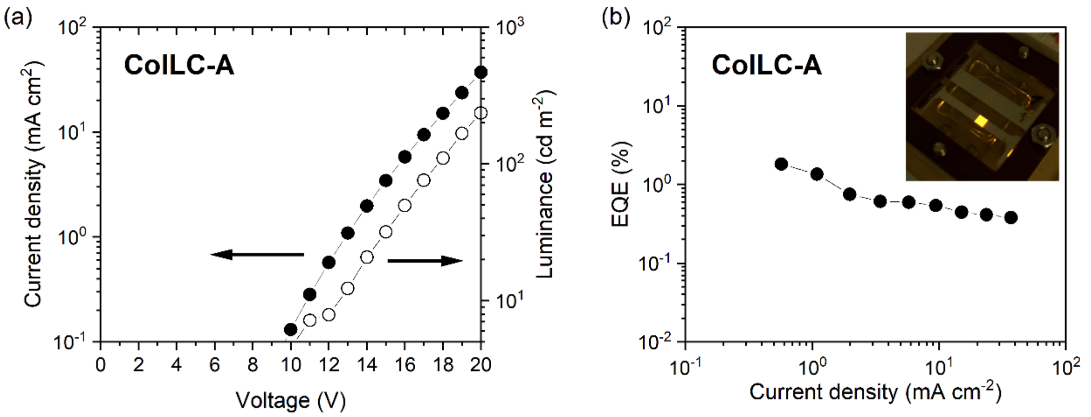

Figure S20: OLED device characteristics using **ColLC-A** as emissive layer: a) current density-voltage and luminance; b) external quantum efficiency (EQE) *vs.* current density. Inset: photograph of an operating device.

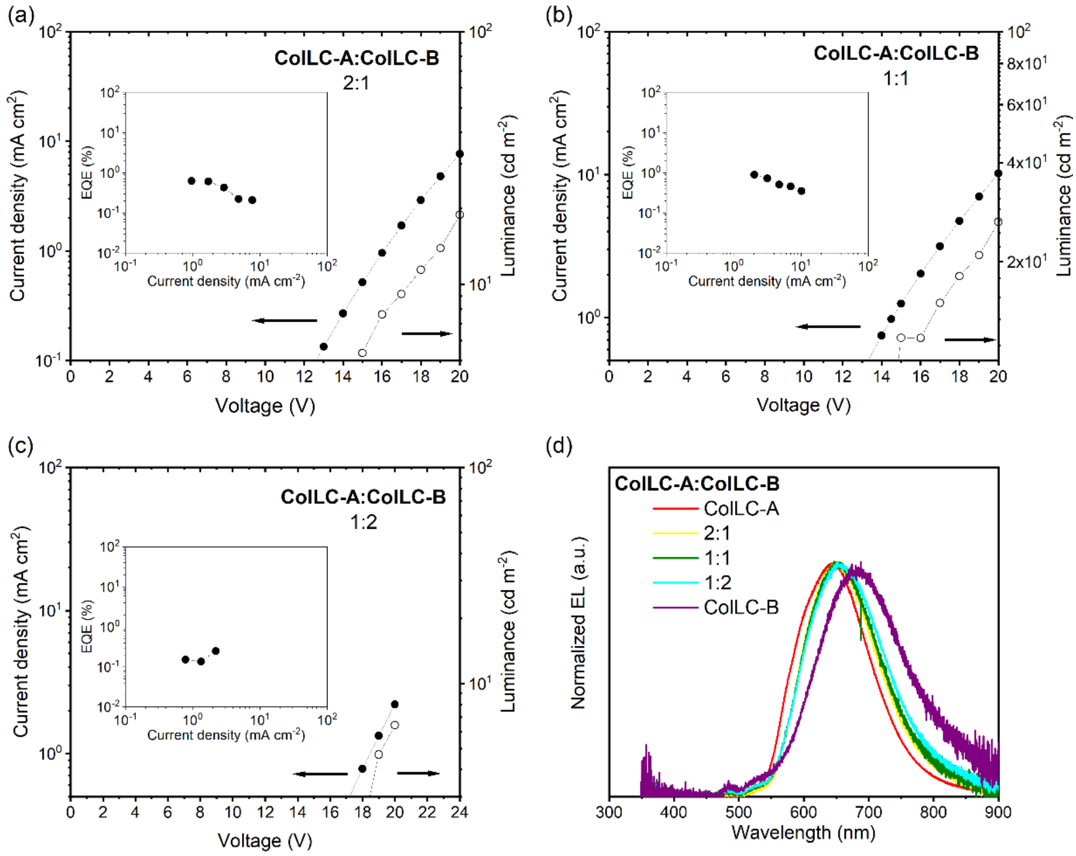

Figure S21: OLED device characteristics: current density *vs.* voltage and luminance, insets: EQE *vs.* current density, using mixtures of **ColLC-A:ColLC-B** in the EML with ratios of (a) 2:1; (b) 1:1; (c) 1:2 w/w. (d) Electroluminescence (EL) spectra at 20 V of devices with **ColLC-A** and blends of **ColLC-A** (2:1; 1:1; 1:2) as well as neat **ColLC-B** as the emissive layer. Device structure was ITO | Al4083 (30 nm) | PVKH (12 nm) | EML (30 nm) | PO-T2T (50 nm) | LiF (0.8 nm) | Al (100 nm) in all devices except for **ColLC-B** where it was ITO | MoO_x (10 nm) | PVKH (12 nm) | **ColLC-B** (30 nm) | PO-T2T (50 nm) | LiF (0.8 nm) | Al (100 nm).

Table S4: Summary of electroluminescent properties of OLED devices. Device structure: ITO | Al4083 (30 nm) | PVKH (12 nm) | EML (30 nm) | PO-T2T (50 nm) | LiF (0.8 nm) | Al (100 nm)

Ratio of ColLC-A:ColLC-B in EML	**[a]V_{ON} (V)**	**[b]L_{max} (cd m^{-2})**	**[c]λ_{max} (nm)**	**[d]CIE 1931 (x; y)**	**[e]$\eta_{ext., max}$ (%)**
**100% ColLC-A**	10	235	646	0.57;0.41	1.8
**2:1**	14	19	647	0.61;0.41	0.6
**1:1**	14	26	646	0.41;0.30	0.9
**1:2**	19	6	652	0.67;0.75	0.2

[a]Turn-on voltage at 5 cd m^{-2}. [b]Maximum luminance. [c]Electroluminescence spectrum maxima. [d]Color coordinates at maximum brightness as defined in International Commission on Illumination color space CIE 1931. [e]Maximum external quantum efficiency.

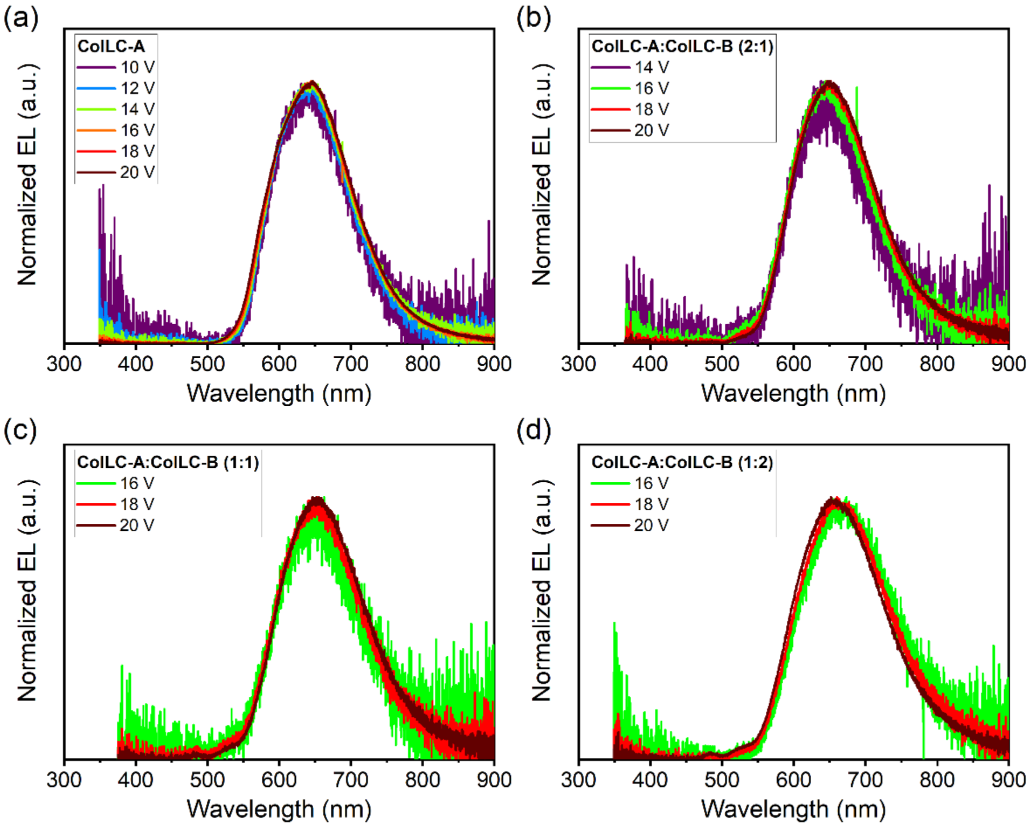

Figure S22: Electroluminescent spectra of OLED devices, using (a) **ColLC-A** and mixtures of **ColLC-A:ColLC-B** in the EML with ratios of (b) 2:1; (c) 1:1; (d) 1:2 m/m at different voltages. Device structure: ITO | Al4083 (30 nm) | PVKH (12 nm) | EML (30 nm) | PO-T2T (50 nm) | LiF (0.8 nm) | Al (100 nm).

Table S5: Non-doped solution-processed OLED devices using columnar liquid crystal as the emissive layer.

[a]*Device structure*	λ_{max} *(nm)*	*CIE 1931 (x; y)*	L_{max} *(cd m^{-2})*	$\eta_{ext.,max}$ *(%)*	*Reference*
ITO/PEDOT/**12**/TPBI(20 nm)/ LiF(10 nm)/Al (100 nm).	582	0.43;0.42	4.5	-	[4]
ITO/NPB(70nm)/ **PTCTE** (110nm)/ LiF(1 nm)/Al (100 nm).	620	-	-	0.25	[5]
ITO (125 nm)/PEDOT: PSS (35 nm)/**PQ10** (20 nm)/TPBi (35 nm)/LiF (1 nm)/ Al (100 nm)	550	0.39;0.48	480	0.2	[6]
ITO/PEDOT:PSS(35nm)/ **Hpz-3C12** (20 nm)/TPBi/ LiF(1 nm)/Al (100 nm)	Blue	-	5	-	[7]
ITO/ PEDOT:PSS (35 nm)/ **Emitter** (25nm)/ TPBi (40 nm)/ LiF (1.5 nm)/ Al (150 nm)	Green	-	3-7	-	[8]
ITO (120 nm)/ PEDOT: PSS (50 nm)/ **7a**[1] (80 nm)/ LiF (1 nm)/ Al (120 nm).	495	0.15;0.36	76	0.09	[9]
ITO/PEDOT:PSS/**3a**/TPBi /LiF/Al	Green	-	7	-	[10]

[a]For explanation of acronyms used see original articles.

4 References

(1) de Sa Pereira, D.; Monkman, A. P.; Data, P. Production and Characterization of Vacuum Deposited Organic Light Emitting Diodes. *J. Vis. Exp.* **2018**, No. 141.

(2) Belarmino Cabral, M. G.; Pereira de Oliveira Santos, D. M.; Cristiano, R.; Gallardo, H.; Bentaleb, A.; Hillard, E. A.; Durola, F.; Bock, H. From 1,4-Phenylenebis(Phenylmaleate) to a Room-Temperature Liquid-Crystalline Benzo[Ghi]Perylene Diimide. *Chempluschem* **2017**, *82* (3), 342–346.

(3) Kelber, J.; Achard, M. F.; Garreau-De Bonneval, B.; Bock, H. Columnar Benzoperylene-Hexa- and Tetracarboxylic Imides and Esters: Synthesis, Mesophase Stabilisation and Observation of Charge-Transfer Interactions between Electron-Donating Esters and Electron-Accepting Imides. *Chem. - A Eur. J.* **2011**, *17* (29), 8145–8155.

(4) Jiang, S.; Qiu, J.; Chen, Y.; Guo, H.; Yang, F. Luminescent Columnar Liquid Crystals Based on AIE Tetraphenylethylene with Hydrazone Groups Bearing Multiple Alkyl Chains. *Dye. Pigment.* **2018**, *159* (May), 533–541.

(5) Keum, C.; Becker, D.; Archer, E.; Bock, H.; Kitzerow, H.; Gather, M. C.; Murawski, C. Organic Light-Emitting Diodes Based on a Columnar Liquid-Crystalline Perylene Emitter. *Adv. Opt. Mater.* **2020**, *8* (17), 2000414.

(6) Vishwakarma, V. K.; Nath, S.; Gupta, M.; Dubey, D. K.; Swayamprabha, S. S.; Jou, J.-H.; Pal, S. K.; Sudhakar, A. A. Room-Temperature Columnar Liquid Crystalline Materials Based on Pyrazino[2,3-g]Quinoxaline for Bright Green Organic Light-Emitting Diodes. *ACS Appl. Electron. Mater.* **2019**, *1* (9), 1959–1969.

(7) Bala, I.; Ming, L.; Yadav, R. A. K.; De, J.; Dubey, D. K.; Kumar, S.; Singh, H.; Jou, J.; Kailasam, K.; Pal, S. K. Deep-Blue OLED Fabrication from Heptazine Columnar Liquid Crystal Based AIE-Active Sky-Blue Emitter. *ChemistrySelect* **2018**, *3* (27), 7771–7777.

(8) De, J.; M. M., A. H.; Yadav, R. A. K.; Gupta, S. P.; Bala, I.; Chawla, P.; Kesavan, K. K.; Jou, J.-H.; Pal, S. K. AIE-Active Mechanoluminescent Discotic Liquid Crystals for Applications in OLEDs and Bio-Imaging. *Chem. Commun.* **2020**, *56* (91), 14279–14282.

(9) Sharma, V. S.; Sharma, A. S.; Agarwal, N. K.; Shah, P. A.; Shrivastav, P. S. Self-Assembled Blue-Light Emitting Materials for Their Liquid Crystalline and OLED Applications: From a

Simple Molecular Design to Supramolecular Materials. *Mol. Syst. Des. Eng.* **2020**, *5* (10), 1691–1705.

(10) Bala, I.; Singh, N.; Yadav, R. A. K.; De, J.; Gupta, S. P.; Singh, D. P.; Dubey, D. K.; Jou, J.-H.; Douali, R.; Pal, S. K. Room Temperature Perylene Based Columnar Liquid Crystals as Solid-State Fluorescent Emitters in Solution-Processable Organic Light-Emitting Diodes. *J. Mater. Chem. C* **2020**, *8* (36), 12485–12494.
